# Supplementary figures and images for: AMSF: attention-based multi-view slice fusion for early diagnosis of Alzheimer’s disease (part 2 of 4)
Source: PeerJ Comput Sci. 2023 Nov 23;9:e1706. doi: 10.7717/peerj-cs.1706 (PMC10703093; doi:10.7717/peerj-cs.1706)

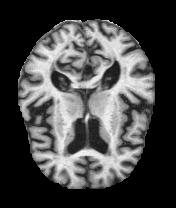

Supplement: Supplemental Information 1 [file peerj-cs-09-1706-s001.zip › MildDemented/mildDem322.jpg]

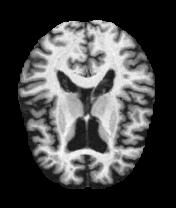

Supplement: Supplemental Information 1 [file peerj-cs-09-1706-s001.zip › MildDemented/mildDem444.jpg]

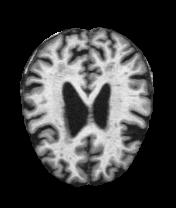

Supplement: Supplemental Information 1 [file peerj-cs-09-1706-s001.zip › MildDemented/32 (27).jpg]

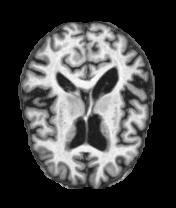

Supplement: Supplemental Information 1 [file peerj-cs-09-1706-s001.zip › MildDemented/mildDem478.jpg]

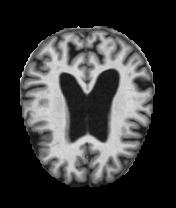

Supplement: Supplemental Information 1 [file peerj-cs-09-1706-s001.zip › MildDemented/29 (21).jpg]

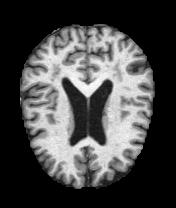

Supplement: Supplemental Information 1 [file peerj-cs-09-1706-s001.zip › MildDemented/mildDem691.jpg]

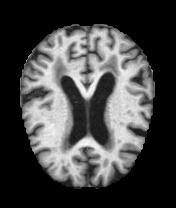

Supplement: Supplemental Information 1 [file peerj-cs-09-1706-s001.zip › MildDemented/mildDem685.jpg]

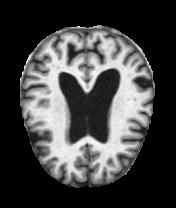

Supplement: Supplemental Information 1 [file peerj-cs-09-1706-s001.zip › MildDemented/28 (21).jpg]

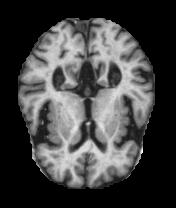

Supplement: Supplemental Information 1 [file peerj-cs-09-1706-s001.zip › MildDemented/mildDem134.jpg]

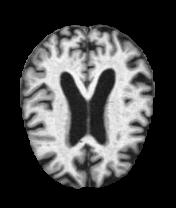

Supplement: Supplemental Information 1 [file peerj-cs-09-1706-s001.zip › MildDemented/26 (19).jpg]

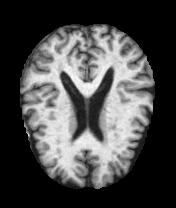

Supplement: Supplemental Information 1 [file peerj-cs-09-1706-s001.zip › MildDemented/mildDem652.jpg]

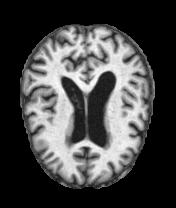

Supplement: Supplemental Information 1 [file peerj-cs-09-1706-s001.zip › MildDemented/mildDem646.jpg]

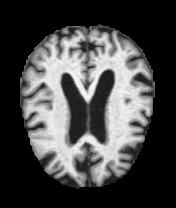

Supplement: Supplemental Information 1 [file peerj-cs-09-1706-s001.zip › MildDemented/27 (19).jpg]

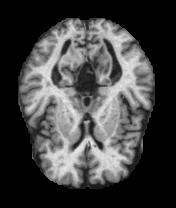

Supplement: Supplemental Information 1 [file peerj-cs-09-1706-s001.zip › MildDemented/mildDem120.jpg]

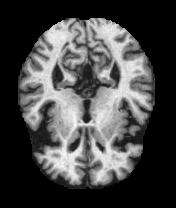

Supplement: Supplemental Information 1 [file peerj-cs-09-1706-s001.zip › MildDemented/mildDem108.jpg]

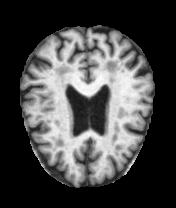

Supplement: Supplemental Information 1 [file peerj-cs-09-1706-s001.zip › MildDemented/28 (5).jpg]

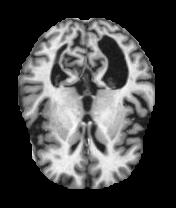

Supplement: Supplemental Information 1 [file peerj-cs-09-1706-s001.zip › MildDemented/mildDem124.jpg]

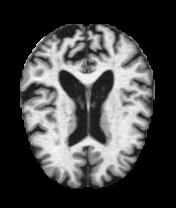

Supplement: Supplemental Information 1 [file peerj-cs-09-1706-s001.zip › MildDemented/mildDem642.jpg]

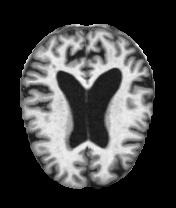

Supplement: Supplemental Information 1 [file peerj-cs-09-1706-s001.zip › MildDemented/mildDem656.jpg]

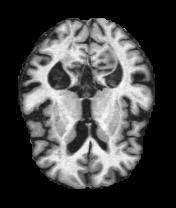

Supplement: Supplemental Information 1 [file peerj-cs-09-1706-s001.zip › MildDemented/mildDem130.jpg]

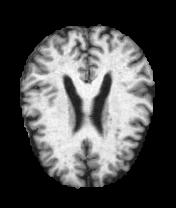

Supplement: Supplemental Information 1 [file peerj-cs-09-1706-s001.zip › MildDemented/27 (18).jpg]

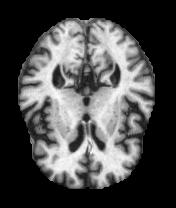

Supplement: Supplemental Information 1 [file peerj-cs-09-1706-s001.zip › MildDemented/mildDem118.jpg]

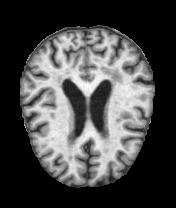

Supplement: Supplemental Information 1 [file peerj-cs-09-1706-s001.zip › MildDemented/28 (4).jpg]

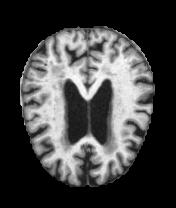

Supplement: Supplemental Information 1 [file peerj-cs-09-1706-s001.zip › MildDemented/29 (20).jpg]

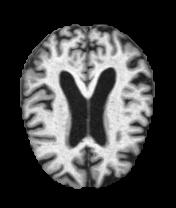

Supplement: Supplemental Information 1 [file peerj-cs-09-1706-s001.zip › MildDemented/mildDem681.jpg]

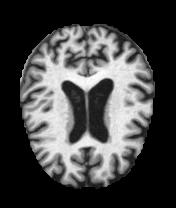

Supplement: Supplemental Information 1 [file peerj-cs-09-1706-s001.zip › MildDemented/mildDem695.jpg]

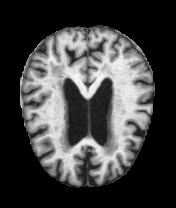

Supplement: Supplemental Information 1 [file peerj-cs-09-1706-s001.zip › MildDemented/28 (20).jpg]

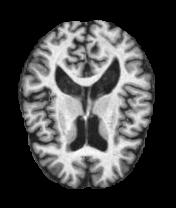

Supplement: Supplemental Information 1 [file peerj-cs-09-1706-s001.zip › MildDemented/mildDem440.jpg]

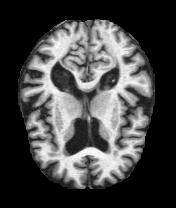

Supplement: Supplemental Information 1 [file peerj-cs-09-1706-s001.zip › MildDemented/mildDem326.jpg]

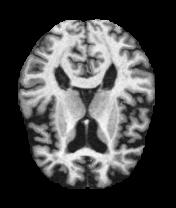

Supplement: Supplemental Information 1 [file peerj-cs-09-1706-s001.zip › MildDemented/mildDem332.jpg]

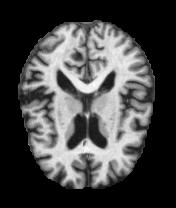

Supplement: Supplemental Information 1 [file peerj-cs-09-1706-s001.zip › MildDemented/mildDem454.jpg]

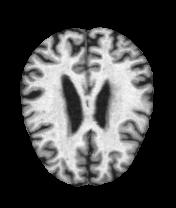

Supplement: Supplemental Information 1 [file peerj-cs-09-1706-s001.zip › MildDemented/32 (26).jpg]

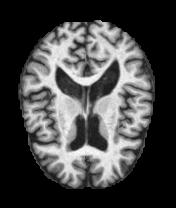

Supplement: Supplemental Information 1 [file peerj-cs-09-1706-s001.zip › MildDemented/mildDem468.jpg]

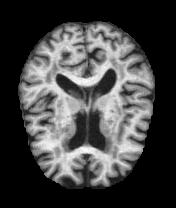

Supplement: Supplemental Information 1 [file peerj-cs-09-1706-s001.zip › MildDemented/mildDem483.jpg]

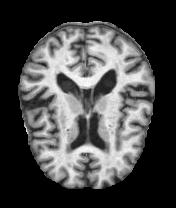

Supplement: Supplemental Information 1 [file peerj-cs-09-1706-s001.zip › MildDemented/mildDem497.jpg]

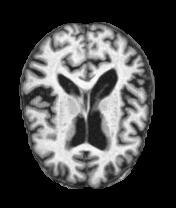

Supplement: Supplemental Information 1 [file peerj-cs-09-1706-s001.zip › MildDemented/mildDem534.jpg]

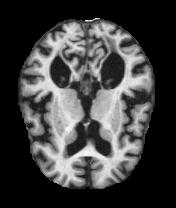

Supplement: Supplemental Information 1 [file peerj-cs-09-1706-s001.zip › MildDemented/mildDem252.jpg]

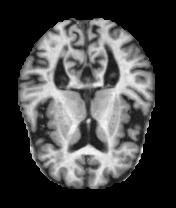

Supplement: Supplemental Information 1 [file peerj-cs-09-1706-s001.zip › MildDemented/mildDem246.jpg]

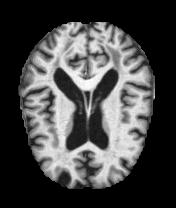

Supplement: Supplemental Information 1 [file peerj-cs-09-1706-s001.zip › MildDemented/mildDem520.jpg]

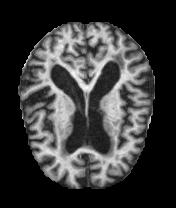

Supplement: Supplemental Information 1 [file peerj-cs-09-1706-s001.zip › MildDemented/mildDem508.jpg]

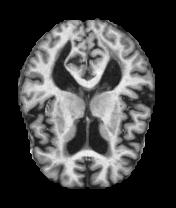

Supplement: Supplemental Information 1 [file peerj-cs-09-1706-s001.zip › MildDemented/mildDem291.jpg]

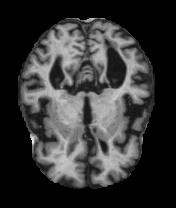

Supplement: Supplemental Information 1 [file peerj-cs-09-1706-s001.zip › MildDemented/mildDem28.jpg]

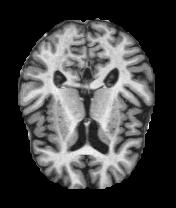

Supplement: Supplemental Information 1 [file peerj-cs-09-1706-s001.zip › MildDemented/mildDem285.jpg]

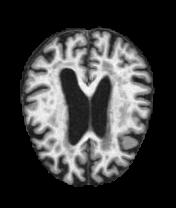

Supplement: Supplemental Information 1 [file peerj-cs-09-1706-s001.zip › MildDemented/31 (8).jpg]

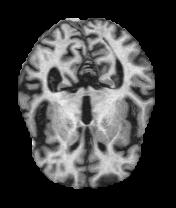

Supplement: Supplemental Information 1 [file peerj-cs-09-1706-s001.zip › MildDemented/mildDem14.jpg]

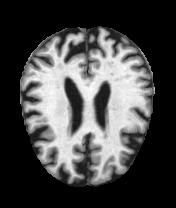

Supplement: Supplemental Information 1 [file peerj-cs-09-1706-s001.zip › MildDemented/30 (9).jpg]

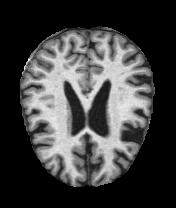

Supplement: Supplemental Information 1 [file peerj-cs-09-1706-s001.zip › MildDemented/30 (15).jpg]

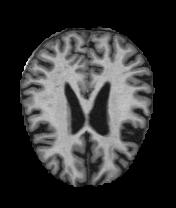

Supplement: Supplemental Information 1 [file peerj-cs-09-1706-s001.zip › MildDemented/31 (15).jpg]

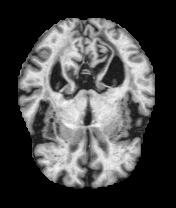

Supplement: Supplemental Information 1 [file peerj-cs-09-1706-s001.zip › MildDemented/mildDem15.jpg]

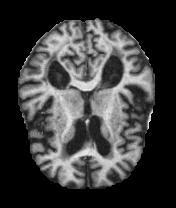

Supplement: Supplemental Information 1 [file peerj-cs-09-1706-s001.zip › MildDemented/mildDem284.jpg]

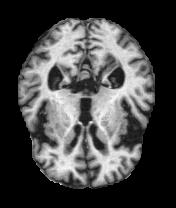

Supplement: Supplemental Information 1 [file peerj-cs-09-1706-s001.zip › MildDemented/mildDem29.jpg]

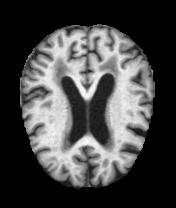

Supplement: Supplemental Information 1 [file peerj-cs-09-1706-s001.zip › MildDemented/26 (22).jpg]

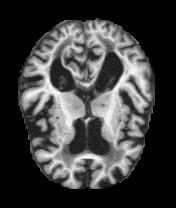

Supplement: Supplemental Information 1 [file peerj-cs-09-1706-s001.zip › MildDemented/mildDem290.jpg]

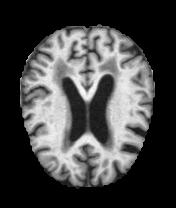

Supplement: Supplemental Information 1 [file peerj-cs-09-1706-s001.zip › MildDemented/27 (22).jpg]

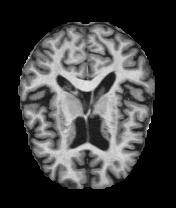

Supplement: Supplemental Information 1 [file peerj-cs-09-1706-s001.zip › MildDemented/mildDem509.jpg]

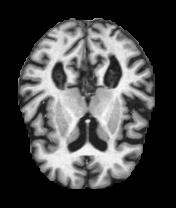

Supplement: Supplemental Information 1 [file peerj-cs-09-1706-s001.zip › MildDemented/mildDem247.jpg]

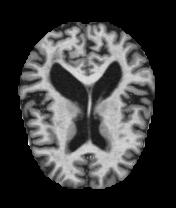

Supplement: Supplemental Information 1 [file peerj-cs-09-1706-s001.zip › MildDemented/mildDem521.jpg]

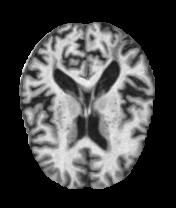

Supplement: Supplemental Information 1 [file peerj-cs-09-1706-s001.zip › MildDemented/mildDem535.jpg]

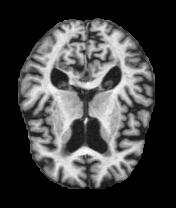

Supplement: Supplemental Information 1 [file peerj-cs-09-1706-s001.zip › MildDemented/mildDem253.jpg]

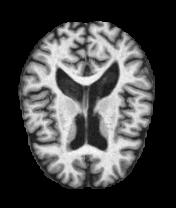

Supplement: Supplemental Information 1 [file peerj-cs-09-1706-s001.zip › MildDemented/mildDem496.jpg]

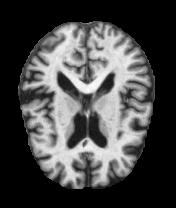

Supplement: Supplemental Information 1 [file peerj-cs-09-1706-s001.zip › MildDemented/mildDem482.jpg]

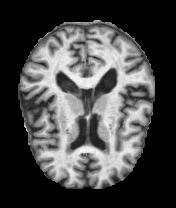

Supplement: Supplemental Information 1 [file peerj-cs-09-1706-s001.zip › MildDemented/mildDem469.jpg]

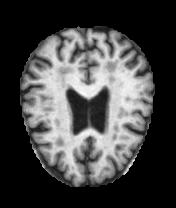

Supplement: Supplemental Information 1 [file peerj-cs-09-1706-s001.zip › MildDemented/29 (5).jpg]

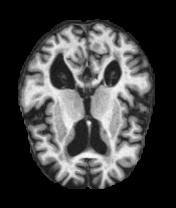

Supplement: Supplemental Information 1 [file peerj-cs-09-1706-s001.zip › MildDemented/mildDem333.jpg]

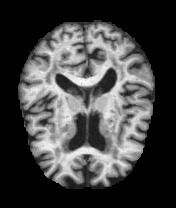

Supplement: Supplemental Information 1 [file peerj-cs-09-1706-s001.zip › MildDemented/mildDem455.jpg]

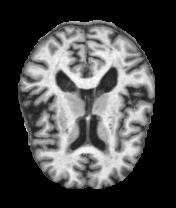

Supplement: Supplemental Information 1 [file peerj-cs-09-1706-s001.zip › MildDemented/mildDem441.jpg]

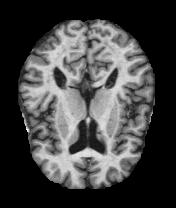

Supplement: Supplemental Information 1 [file peerj-cs-09-1706-s001.zip › MildDemented/mildDem327.jpg]

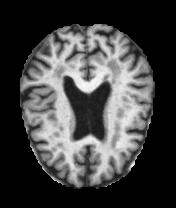

Supplement: Supplemental Information 1 [file peerj-cs-09-1706-s001.zip › MildDemented/mildDem694.jpg]

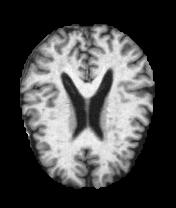

Supplement: Supplemental Information 1 [file peerj-cs-09-1706-s001.zip › MildDemented/mildDem680.jpg]

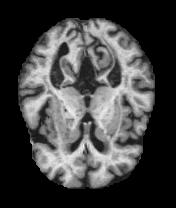

Supplement: Supplemental Information 1 [file peerj-cs-09-1706-s001.zip › MildDemented/mildDem119.jpg]

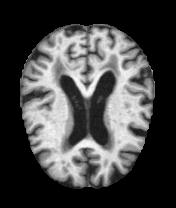

Supplement: Supplemental Information 1 [file peerj-cs-09-1706-s001.zip › MildDemented/mildDem657.jpg]

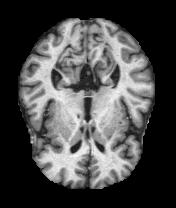

Supplement: Supplemental Information 1 [file peerj-cs-09-1706-s001.zip › MildDemented/mildDem131.jpg]

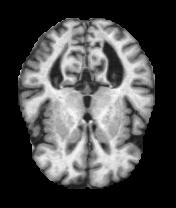

Supplement: Supplemental Information 1 [file peerj-cs-09-1706-s001.zip › MildDemented/mildDem125.jpg]

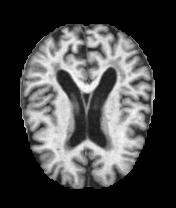

Supplement: Supplemental Information 1 [file peerj-cs-09-1706-s001.zip › MildDemented/mildDem643.jpg]

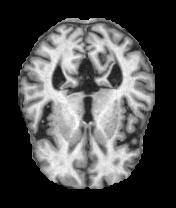

Supplement: Supplemental Information 1 [file peerj-cs-09-1706-s001.zip › MildDemented/mildDem133.jpg]

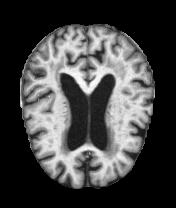

Supplement: Supplemental Information 1 [file peerj-cs-09-1706-s001.zip › MildDemented/mildDem655.jpg]

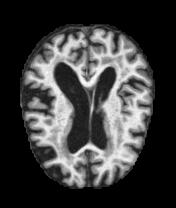

Supplement: Supplemental Information 1 [file peerj-cs-09-1706-s001.zip › MildDemented/mildDem641.jpg]

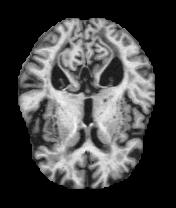

Supplement: Supplemental Information 1 [file peerj-cs-09-1706-s001.zip › MildDemented/mildDem127.jpg]

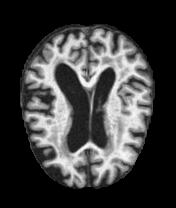

Supplement: Supplemental Information 1 [file peerj-cs-09-1706-s001.zip › MildDemented/mildDem669.jpg]

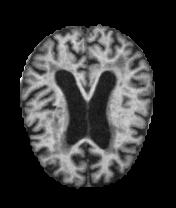

Supplement: Supplemental Information 1 [file peerj-cs-09-1706-s001.zip › MildDemented/27 (14).jpg]

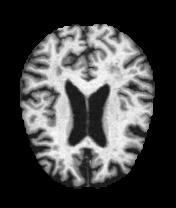

Supplement: Supplemental Information 1 [file peerj-cs-09-1706-s001.zip › MildDemented/mildDem696.jpg]

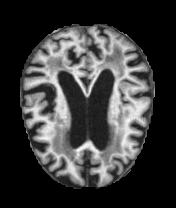

Supplement: Supplemental Information 1 [file peerj-cs-09-1706-s001.zip › MildDemented/mildDem682.jpg]

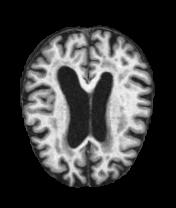

Supplement: Supplemental Information 1 [file peerj-cs-09-1706-s001.zip › MildDemented/28 (8).jpg]

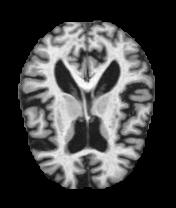

Supplement: Supplemental Information 1 [file peerj-cs-09-1706-s001.zip › MildDemented/mildDem457.jpg]

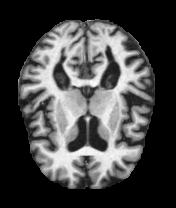

Supplement: Supplemental Information 1 [file peerj-cs-09-1706-s001.zip › MildDemented/mildDem331.jpg]

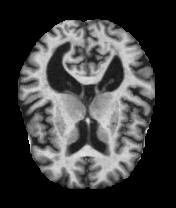

Supplement: Supplemental Information 1 [file peerj-cs-09-1706-s001.zip › MildDemented/mildDem325.jpg]

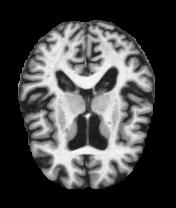

Supplement: Supplemental Information 1 [file peerj-cs-09-1706-s001.zip › MildDemented/mildDem443.jpg]

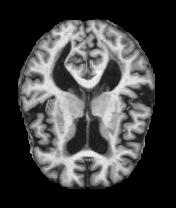

Supplement: Supplemental Information 1 [file peerj-cs-09-1706-s001.zip › MildDemented/mildDem319.jpg]

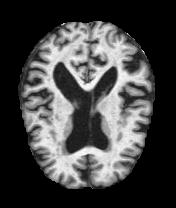

Supplement: Supplemental Information 1 [file peerj-cs-09-1706-s001.zip › MildDemented/mildDem494.jpg]

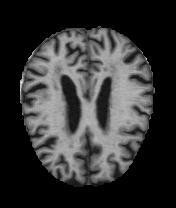

Supplement: Supplemental Information 1 [file peerj-cs-09-1706-s001.zip › MildDemented/31 (23).jpg]

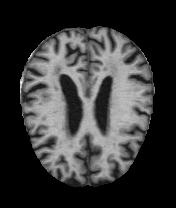

Supplement: Supplemental Information 1 [file peerj-cs-09-1706-s001.zip › MildDemented/30 (23).jpg]

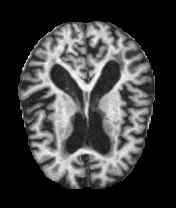

Supplement: Supplemental Information 1 [file peerj-cs-09-1706-s001.zip › MildDemented/mildDem480.jpg]

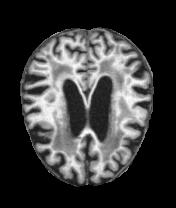

Supplement: Supplemental Information 1 [file peerj-cs-09-1706-s001.zip › MildDemented/32 (2).jpg]

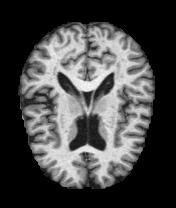

Supplement: Supplemental Information 1 [file peerj-cs-09-1706-s001.zip › MildDemented/mildDem523.jpg]

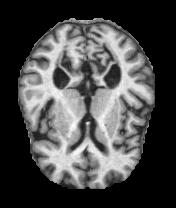

Supplement: Supplemental Information 1 [file peerj-cs-09-1706-s001.zip › MildDemented/mildDem245.jpg]

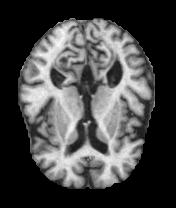

Supplement: Supplemental Information 1 [file peerj-cs-09-1706-s001.zip › MildDemented/mildDem251.jpg]

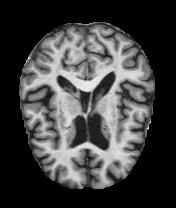

Supplement: Supplemental Information 1 [file peerj-cs-09-1706-s001.zip › MildDemented/mildDem537.jpg]

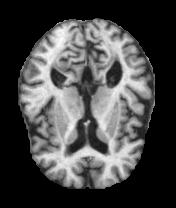

Supplement: Supplemental Information 1 [file peerj-cs-09-1706-s001.zip › MildDemented/mildDem279.jpg]

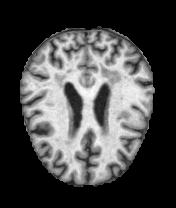

Supplement: Supplemental Information 1 [file peerj-cs-09-1706-s001.zip › MildDemented/31 (4).jpg]

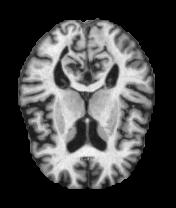

Supplement: Supplemental Information 1 [file peerj-cs-09-1706-s001.zip › MildDemented/mildDem286.jpg]
