# Supplementary figures and images for: AMSF: attention-based multi-view slice fusion for early diagnosis of Alzheimer’s disease (part 1 of 4)
Source: PeerJ Comput Sci. 2023 Nov 23;9:e1706. doi: 10.7717/peerj-cs.1706 (PMC10703093; doi:10.7717/peerj-cs.1706)

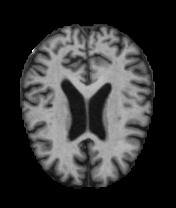

Supplement: Supplemental Information 1 [file peerj-cs-09-1706-s001.zip › MildDemented/mildDem678.jpg]

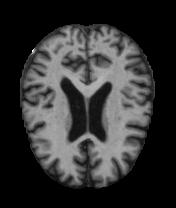

Supplement: Supplemental Information 1 [file peerj-cs-09-1706-s001.zip › MildDemented/mildDem650.jpg]

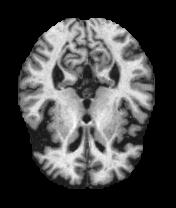

Supplement: Supplemental Information 1 [file peerj-cs-09-1706-s001.zip › MildDemented/mildDem136.jpg]

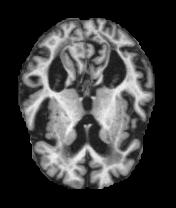

Supplement: Supplemental Information 1 [file peerj-cs-09-1706-s001.zip › MildDemented/mildDem122.jpg]

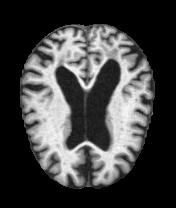

Supplement: Supplemental Information 1 [file peerj-cs-09-1706-s001.zip › MildDemented/mildDem644.jpg]

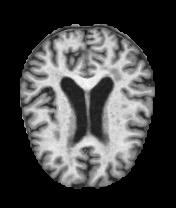

Supplement: Supplemental Information 1 [file peerj-cs-09-1706-s001.zip › MildDemented/mildDem693.jpg]

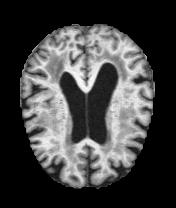

Supplement: Supplemental Information 1 [file peerj-cs-09-1706-s001.zip › MildDemented/mildDem687.jpg]

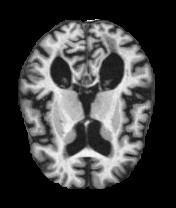

Supplement: Supplemental Information 1 [file peerj-cs-09-1706-s001.zip › MildDemented/mildDem308.jpg]

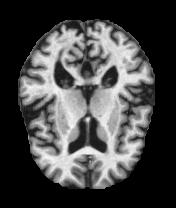

Supplement: Supplemental Information 1 [file peerj-cs-09-1706-s001.zip › MildDemented/mildDem334.jpg]

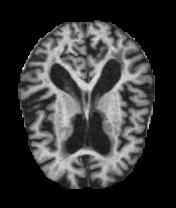

Supplement: Supplemental Information 1 [file peerj-cs-09-1706-s001.zip › MildDemented/mildDem452.jpg]

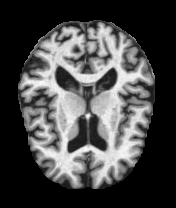

Supplement: Supplemental Information 1 [file peerj-cs-09-1706-s001.zip › MildDemented/mildDem446.jpg]

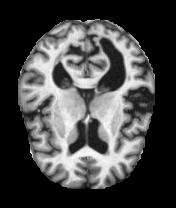

Supplement: Supplemental Information 1 [file peerj-cs-09-1706-s001.zip › MildDemented/mildDem320.jpg]

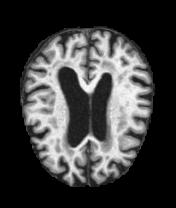

Supplement: Supplemental Information 1 [file peerj-cs-09-1706-s001.zip › MildDemented/29 (8).jpg]

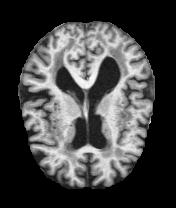

Supplement: Supplemental Information 1 [file peerj-cs-09-1706-s001.zip › MildDemented/mildDem491.jpg]

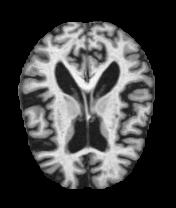

Supplement: Supplemental Information 1 [file peerj-cs-09-1706-s001.zip › MildDemented/mildDem485.jpg]

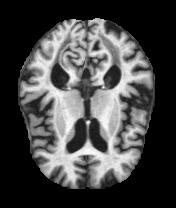

Supplement: Supplemental Information 1 [file peerj-cs-09-1706-s001.zip › MildDemented/mildDem268.jpg]

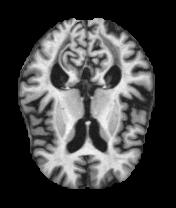

Supplement: Supplemental Information 1 [file peerj-cs-09-1706-s001.zip › MildDemented/mildDem240.jpg]

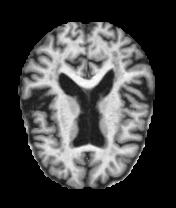

Supplement: Supplemental Information 1 [file peerj-cs-09-1706-s001.zip › MildDemented/mildDem526.jpg]

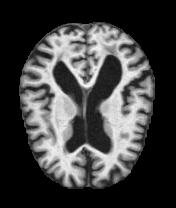

Supplement: Supplemental Information 1 [file peerj-cs-09-1706-s001.zip › MildDemented/mildDem532.jpg]

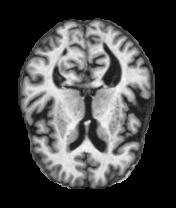

Supplement: Supplemental Information 1 [file peerj-cs-09-1706-s001.zip › MildDemented/mildDem254.jpg]

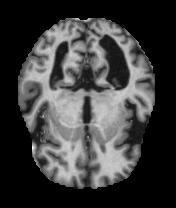

Supplement: Supplemental Information 1 [file peerj-cs-09-1706-s001.zip › MildDemented/mildDem12.jpg]

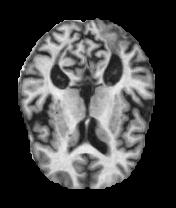

Supplement: Supplemental Information 1 [file peerj-cs-09-1706-s001.zip › MildDemented/mildDem283.jpg]

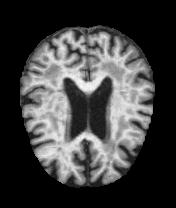

Supplement: Supplemental Information 1 [file peerj-cs-09-1706-s001.zip › MildDemented/28 (17).jpg]

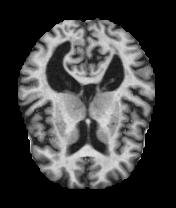

Supplement: Supplemental Information 1 [file peerj-cs-09-1706-s001.zip › MildDemented/mildDem297.jpg]

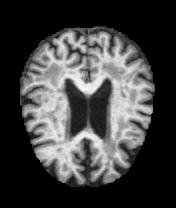

Supplement: Supplemental Information 1 [file peerj-cs-09-1706-s001.zip › MildDemented/29 (17).jpg]

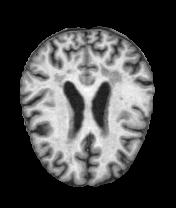

Supplement: Supplemental Information 1 [file peerj-cs-09-1706-s001.zip › MildDemented/30 (4).jpg]

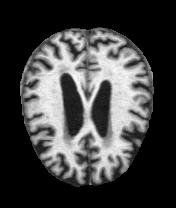

Supplement: Supplemental Information 1 [file peerj-cs-09-1706-s001.zip › MildDemented/32 (11).jpg]

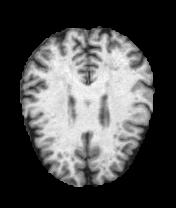

Supplement: Supplemental Information 1 [file peerj-cs-09-1706-s001.zip › MildDemented/31 (18).jpg]

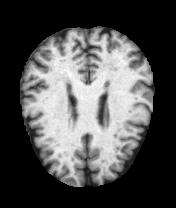

Supplement: Supplemental Information 1 [file peerj-cs-09-1706-s001.zip › MildDemented/30 (18).jpg]

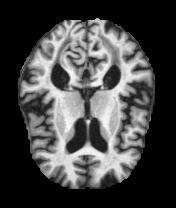

Supplement: Supplemental Information 1 [file peerj-cs-09-1706-s001.zip › MildDemented/mildDem296.jpg]

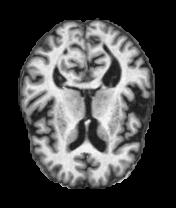

Supplement: Supplemental Information 1 [file peerj-cs-09-1706-s001.zip › MildDemented/mildDem282.jpg]

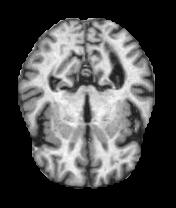

Supplement: Supplemental Information 1 [file peerj-cs-09-1706-s001.zip › MildDemented/mildDem13.jpg]

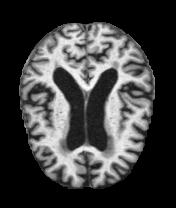

Supplement: Supplemental Information 1 [file peerj-cs-09-1706-s001.zip › MildDemented/mildDem533.jpg]

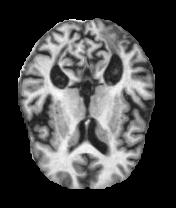

Supplement: Supplemental Information 1 [file peerj-cs-09-1706-s001.zip › MildDemented/mildDem255.jpg]

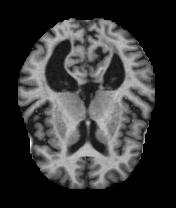

Supplement: Supplemental Information 1 [file peerj-cs-09-1706-s001.zip › MildDemented/mildDem241.jpg]

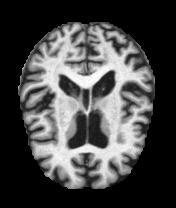

Supplement: Supplemental Information 1 [file peerj-cs-09-1706-s001.zip › MildDemented/mildDem527.jpg]

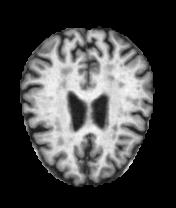

Supplement: Supplemental Information 1 [file peerj-cs-09-1706-s001.zip › MildDemented/31 (5).jpg]

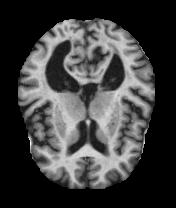

Supplement: Supplemental Information 1 [file peerj-cs-09-1706-s001.zip › MildDemented/mildDem269.jpg]

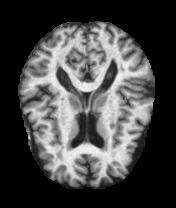

Supplement: Supplemental Information 1 [file peerj-cs-09-1706-s001.zip › MildDemented/mildDem484.jpg]

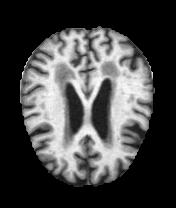

Supplement: Supplemental Information 1 [file peerj-cs-09-1706-s001.zip › MildDemented/31 (22).jpg]

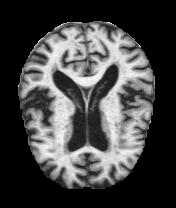

Supplement: Supplemental Information 1 [file peerj-cs-09-1706-s001.zip › MildDemented/mildDem490.jpg]

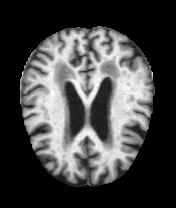

Supplement: Supplemental Information 1 [file peerj-cs-09-1706-s001.zip › MildDemented/30 (22).jpg]

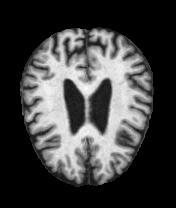

Supplement: Supplemental Information 1 [file peerj-cs-09-1706-s001.zip › MildDemented/32 (3).jpg]

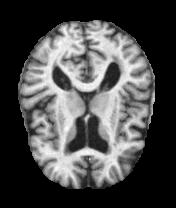

Supplement: Supplemental Information 1 [file peerj-cs-09-1706-s001.zip › MildDemented/mildDem447.jpg]

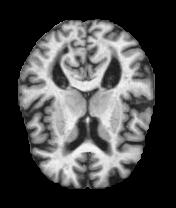

Supplement: Supplemental Information 1 [file peerj-cs-09-1706-s001.zip › MildDemented/mildDem321.jpg]

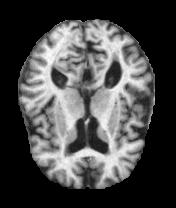

Supplement: Supplemental Information 1 [file peerj-cs-09-1706-s001.zip › MildDemented/mildDem335.jpg]

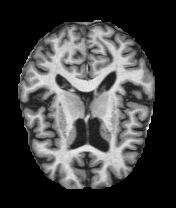

Supplement: Supplemental Information 1 [file peerj-cs-09-1706-s001.zip › MildDemented/mildDem453.jpg]

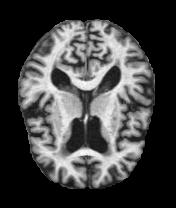

Supplement: Supplemental Information 1 [file peerj-cs-09-1706-s001.zip › MildDemented/mildDem309.jpg]

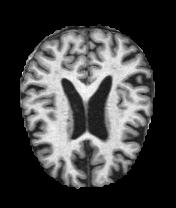

Supplement: Supplemental Information 1 [file peerj-cs-09-1706-s001.zip › MildDemented/27 (15).jpg]

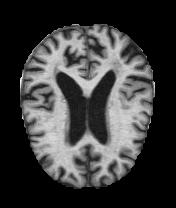

Supplement: Supplemental Information 1 [file peerj-cs-09-1706-s001.zip › MildDemented/mildDem686.jpg]

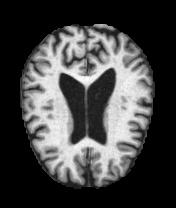

Supplement: Supplemental Information 1 [file peerj-cs-09-1706-s001.zip › MildDemented/mildDem692.jpg]

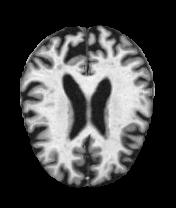

Supplement: Supplemental Information 1 [file peerj-cs-09-1706-s001.zip › MildDemented/28 (9).jpg]

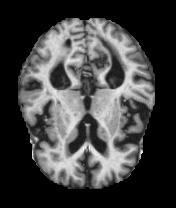

Supplement: Supplemental Information 1 [file peerj-cs-09-1706-s001.zip › MildDemented/mildDem123.jpg]

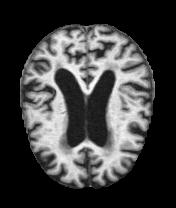

Supplement: Supplemental Information 1 [file peerj-cs-09-1706-s001.zip › MildDemented/mildDem645.jpg]

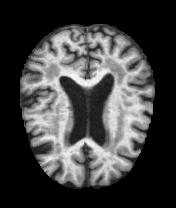

Supplement: Supplemental Information 1 [file peerj-cs-09-1706-s001.zip › MildDemented/mildDem651.jpg]

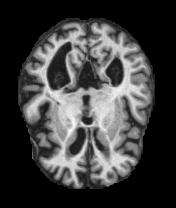

Supplement: Supplemental Information 1 [file peerj-cs-09-1706-s001.zip › MildDemented/mildDem137.jpg]

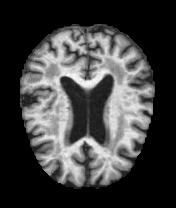

Supplement: Supplemental Information 1 [file peerj-cs-09-1706-s001.zip › MildDemented/mildDem679.jpg]

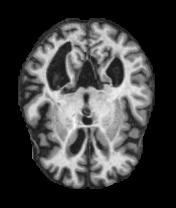

Supplement: Supplemental Information 1 [file peerj-cs-09-1706-s001.zip › MildDemented/mildDem109.jpg]

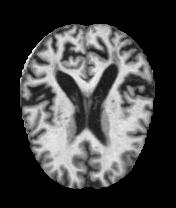

Supplement: Supplemental Information 1 [file peerj-cs-09-1706-s001.zip › MildDemented/mildDem647.jpg]

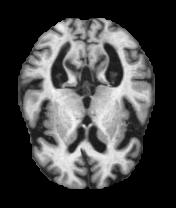

Supplement: Supplemental Information 1 [file peerj-cs-09-1706-s001.zip › MildDemented/mildDem121.jpg]

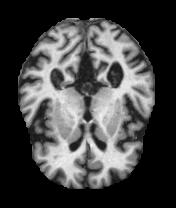

Supplement: Supplemental Information 1 [file peerj-cs-09-1706-s001.zip › MildDemented/mildDem135.jpg]

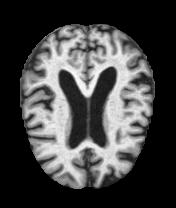

Supplement: Supplemental Information 1 [file peerj-cs-09-1706-s001.zip › MildDemented/mildDem653.jpg]

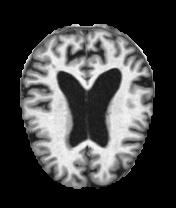

Supplement: Supplemental Information 1 [file peerj-cs-09-1706-s001.zip › MildDemented/mildDem684.jpg]

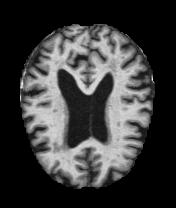

Supplement: Supplemental Information 1 [file peerj-cs-09-1706-s001.zip › MildDemented/mildDem690.jpg]

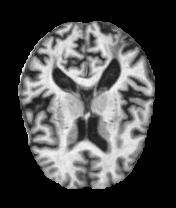

Supplement: Supplemental Information 1 [file peerj-cs-09-1706-s001.zip › MildDemented/mildDem479.jpg]

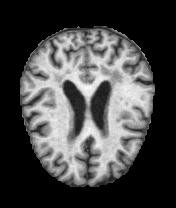

Supplement: Supplemental Information 1 [file peerj-cs-09-1706-s001.zip › MildDemented/29 (4).jpg]

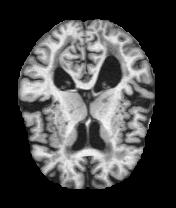

Supplement: Supplemental Information 1 [file peerj-cs-09-1706-s001.zip › MildDemented/mildDem323.jpg]

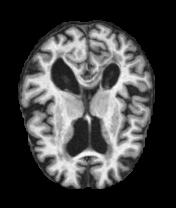

Supplement: Supplemental Information 1 [file peerj-cs-09-1706-s001.zip › MildDemented/mildDem445.jpg]

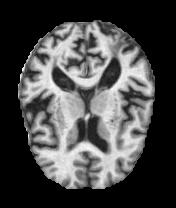

Supplement: Supplemental Information 1 [file peerj-cs-09-1706-s001.zip › MildDemented/mildDem451.jpg]

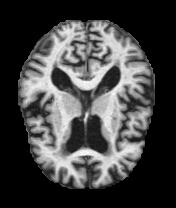

Supplement: Supplemental Information 1 [file peerj-cs-09-1706-s001.zip › MildDemented/mildDem337.jpg]

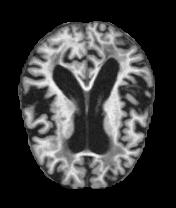

Supplement: Supplemental Information 1 [file peerj-cs-09-1706-s001.zip › MildDemented/mildDem486.jpg]

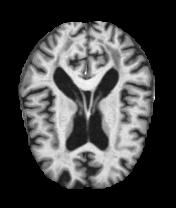

Supplement: Supplemental Information 1 [file peerj-cs-09-1706-s001.zip › MildDemented/mildDem492.jpg]

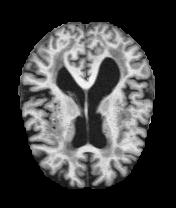

Supplement: Supplemental Information 1 [file peerj-cs-09-1706-s001.zip › MildDemented/mildDem519.jpg]

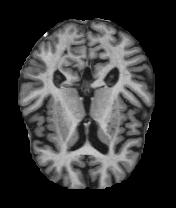

Supplement: Supplemental Information 1 [file peerj-cs-09-1706-s001.zip › MildDemented/mildDem257.jpg]

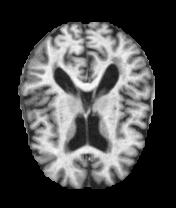

Supplement: Supplemental Information 1 [file peerj-cs-09-1706-s001.zip › MildDemented/mildDem531.jpg]

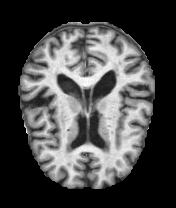

Supplement: Supplemental Information 1 [file peerj-cs-09-1706-s001.zip › MildDemented/mildDem525.jpg]

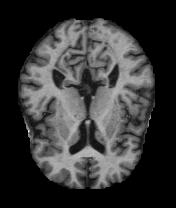

Supplement: Supplemental Information 1 [file peerj-cs-09-1706-s001.zip › MildDemented/mildDem243.jpg]

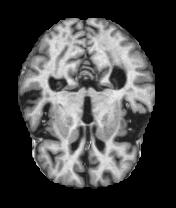

Supplement: Supplemental Information 1 [file peerj-cs-09-1706-s001.zip › MildDemented/mildDem11.jpg]

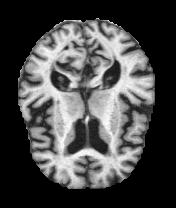

Supplement: Supplemental Information 1 [file peerj-cs-09-1706-s001.zip › MildDemented/mildDem294.jpg]

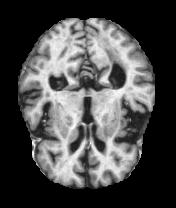

Supplement: Supplemental Information 1 [file peerj-cs-09-1706-s001.zip › MildDemented/mildDem39.jpg]

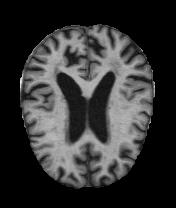

Supplement: Supplemental Information 1 [file peerj-cs-09-1706-s001.zip › MildDemented/26 (23).jpg]

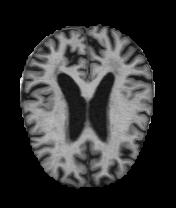

Supplement: Supplemental Information 1 [file peerj-cs-09-1706-s001.zip › MildDemented/27 (23).jpg]

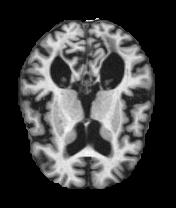

Supplement: Supplemental Information 1 [file peerj-cs-09-1706-s001.zip › MildDemented/mildDem280.jpg]

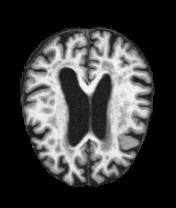

Supplement: Supplemental Information 1 [file peerj-cs-09-1706-s001.zip › MildDemented/30 (8).jpg]

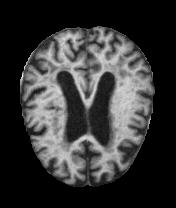

Supplement: Supplemental Information 1 [file peerj-cs-09-1706-s001.zip › MildDemented/30 (14).jpg]

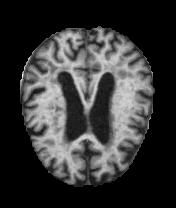

Supplement: Supplemental Information 1 [file peerj-cs-09-1706-s001.zip › MildDemented/31 (14).jpg]

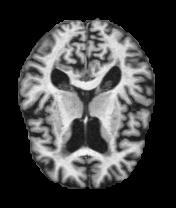

Supplement: Supplemental Information 1 [file peerj-cs-09-1706-s001.zip › MildDemented/mildDem281.jpg]

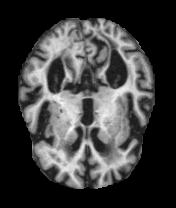

Supplement: Supplemental Information 1 [file peerj-cs-09-1706-s001.zip › MildDemented/mildDem38.jpg]

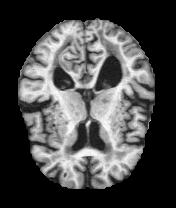

Supplement: Supplemental Information 1 [file peerj-cs-09-1706-s001.zip › MildDemented/mildDem295.jpg]

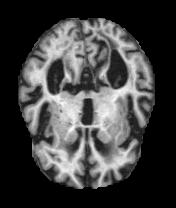

Supplement: Supplemental Information 1 [file peerj-cs-09-1706-s001.zip › MildDemented/mildDem10.jpg]

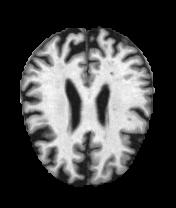

Supplement: Supplemental Information 1 [file peerj-cs-09-1706-s001.zip › MildDemented/31 (9).jpg]

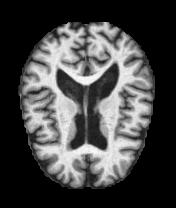

Supplement: Supplemental Information 1 [file peerj-cs-09-1706-s001.zip › MildDemented/mildDem524.jpg]

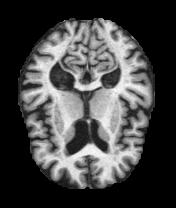

Supplement: Supplemental Information 1 [file peerj-cs-09-1706-s001.zip › MildDemented/mildDem242.jpg]

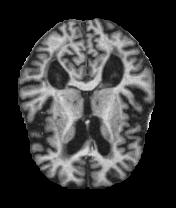

Supplement: Supplemental Information 1 [file peerj-cs-09-1706-s001.zip › MildDemented/mildDem256.jpg]

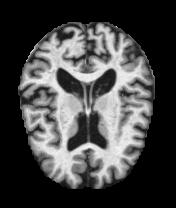

Supplement: Supplemental Information 1 [file peerj-cs-09-1706-s001.zip › MildDemented/mildDem530.jpg]

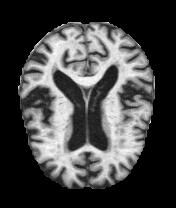

Supplement: Supplemental Information 1 [file peerj-cs-09-1706-s001.zip › MildDemented/mildDem518.jpg]

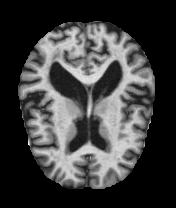

Supplement: Supplemental Information 1 [file peerj-cs-09-1706-s001.zip › MildDemented/mildDem493.jpg]

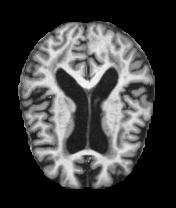

Supplement: Supplemental Information 1 [file peerj-cs-09-1706-s001.zip › MildDemented/mildDem487.jpg]

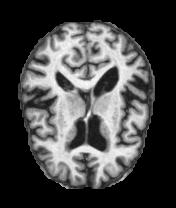

Supplement: Supplemental Information 1 [file peerj-cs-09-1706-s001.zip › MildDemented/mildDem450.jpg]

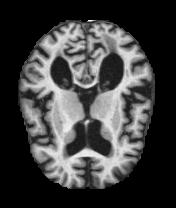

Supplement: Supplemental Information 1 [file peerj-cs-09-1706-s001.zip › MildDemented/mildDem336.jpg]
