# Supplementary figures and images for: AMSF: attention-based multi-view slice fusion for early diagnosis of Alzheimer’s disease (part 4 of 4)
Source: PeerJ Comput Sci. 2023 Nov 23;9:e1706. doi: 10.7717/peerj-cs.1706 (PMC10703093; doi:10.7717/peerj-cs.1706)

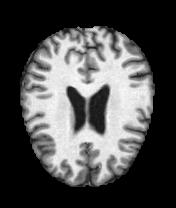

Supplement: Supplemental Information 2 [file peerj-cs-09-1706-s002.zip › NonDemented/29 (8).jpg]

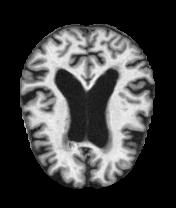

Supplement: Supplemental Information 2 [file peerj-cs-09-1706-s002.zip › NonDemented/nonDem2344.jpg]

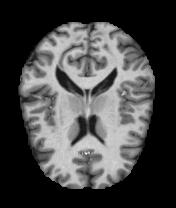

Supplement: Supplemental Information 2 [file peerj-cs-09-1706-s002.zip › NonDemented/nonDem1895.jpg]

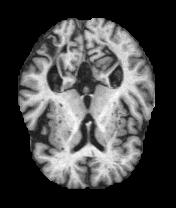

Supplement: Supplemental Information 2 [file peerj-cs-09-1706-s002.zip › NonDemented/nonDem664.jpg]

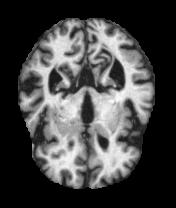

Supplement: Supplemental Information 2 [file peerj-cs-09-1706-s002.zip › NonDemented/nonDem102.jpg]

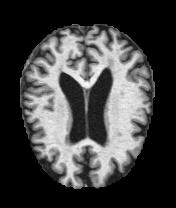

Supplement: Supplemental Information 2 [file peerj-cs-09-1706-s002.zip › NonDemented/nonDem2422.jpg]

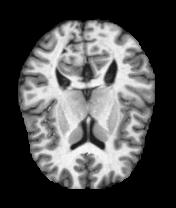

Supplement: Supplemental Information 2 [file peerj-cs-09-1706-s002.zip › NonDemented/nonDem1103.jpg]

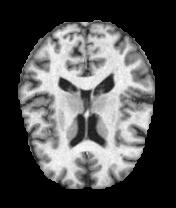

Supplement: Supplemental Information 2 [file peerj-cs-09-1706-s002.zip › NonDemented/nonDem1665.jpg]

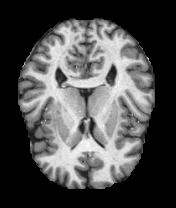

Supplement: Supplemental Information 2 [file peerj-cs-09-1706-s002.zip › NonDemented/nonDem894.jpg]

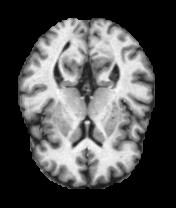

Supplement: Supplemental Information 2 [file peerj-cs-09-1706-s002.zip › NonDemented/nonDem658.jpg]

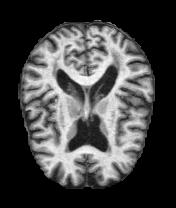

Supplement: Supplemental Information 2 [file peerj-cs-09-1706-s002.zip › NonDemented/nonDem1671.jpg]

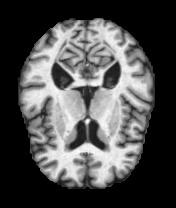

Supplement: Supplemental Information 2 [file peerj-cs-09-1706-s002.zip › NonDemented/nonDem880.jpg]

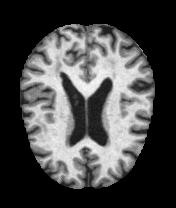

Supplement: Supplemental Information 2 [file peerj-cs-09-1706-s002.zip › NonDemented/nonDem2378.jpg]

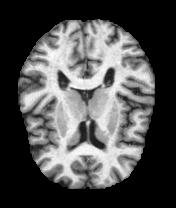

Supplement: Supplemental Information 2 [file peerj-cs-09-1706-s002.zip › NonDemented/nonDem1117.jpg]

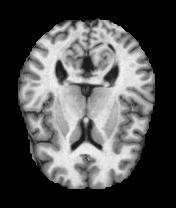

Supplement: Supplemental Information 2 [file peerj-cs-09-1706-s002.zip › NonDemented/nonDem1088.jpg]

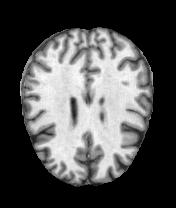

Supplement: Supplemental Information 2 [file peerj-cs-09-1706-s002.zip › NonDemented/32 (46).jpg]

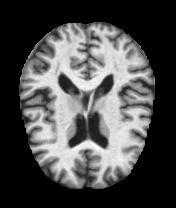

Supplement: Supplemental Information 2 [file peerj-cs-09-1706-s002.zip › NonDemented/nonDem1936.jpg]

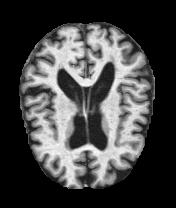

Supplement: Supplemental Information 2 [file peerj-cs-09-1706-s002.zip › NonDemented/nonDem1922.jpg]

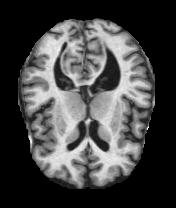

Supplement: Supplemental Information 2 [file peerj-cs-09-1706-s002.zip › NonDemented/nonDem923.jpg]

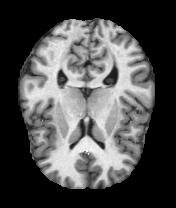

Supplement: Supplemental Information 2 [file peerj-cs-09-1706-s002.zip › NonDemented/nonDem937.jpg]

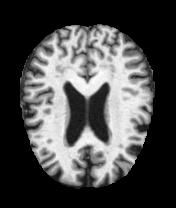

Supplement: Supplemental Information 2 [file peerj-cs-09-1706-s002.zip › NonDemented/nonDem2542.jpg]

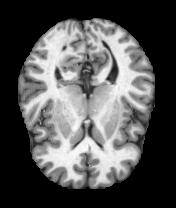

Supplement: Supplemental Information 2 [file peerj-cs-09-1706-s002.zip › NonDemented/nonDem704.jpg]

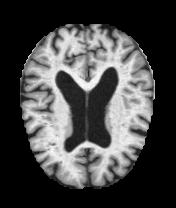

Supplement: Supplemental Information 2 [file peerj-cs-09-1706-s002.zip › NonDemented/nonDem2224.jpg]

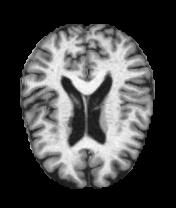

Supplement: Supplemental Information 2 [file peerj-cs-09-1706-s002.zip › NonDemented/nonDem2230.jpg]

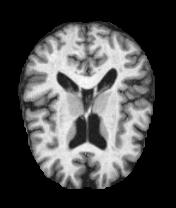

Supplement: Supplemental Information 2 [file peerj-cs-09-1706-s002.zip › NonDemented/nonDem1739.jpg]

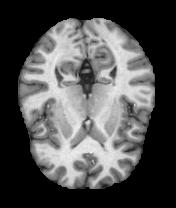

Supplement: Supplemental Information 2 [file peerj-cs-09-1706-s002.zip › NonDemented/nonDem710.jpg]

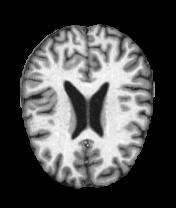

Supplement: Supplemental Information 2 [file peerj-cs-09-1706-s002.zip › NonDemented/nonDem2556.jpg]

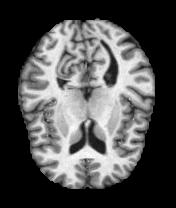

Supplement: Supplemental Information 2 [file peerj-cs-09-1706-s002.zip › NonDemented/nonDem1077.jpg]

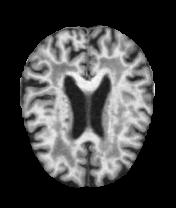

Supplement: Supplemental Information 2 [file peerj-cs-09-1706-s002.zip › NonDemented/28 (17).jpg]

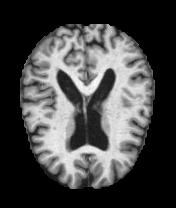

Supplement: Supplemental Information 2 [file peerj-cs-09-1706-s002.zip › NonDemented/nonDem2218.jpg]

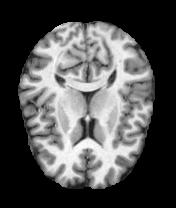

Supplement: Supplemental Information 2 [file peerj-cs-09-1706-s002.zip › NonDemented/nonDem1711.jpg]

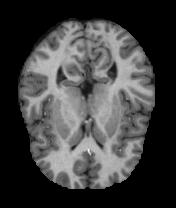

Supplement: Supplemental Information 2 [file peerj-cs-09-1706-s002.zip › NonDemented/nonDem738.jpg]

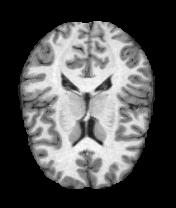

Supplement: Supplemental Information 2 [file peerj-cs-09-1706-s002.zip › NonDemented/nonDem1705.jpg]

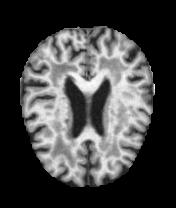

Supplement: Supplemental Information 2 [file peerj-cs-09-1706-s002.zip › NonDemented/29 (17).jpg]

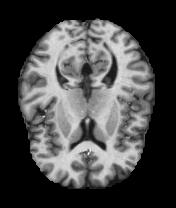

Supplement: Supplemental Information 2 [file peerj-cs-09-1706-s002.zip › NonDemented/nonDem1063.jpg]

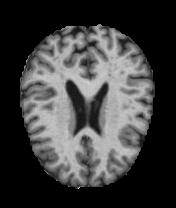

Supplement: Supplemental Information 2 [file peerj-cs-09-1706-s002.zip › NonDemented/27 (97).jpg]

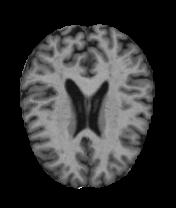

Supplement: Supplemental Information 2 [file peerj-cs-09-1706-s002.zip › NonDemented/26 (97).jpg]

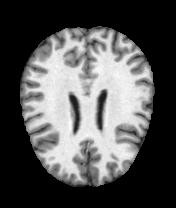

Supplement: Supplemental Information 2 [file peerj-cs-09-1706-s002.zip › NonDemented/30 (4).jpg]

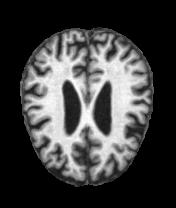

Supplement: Supplemental Information 2 [file peerj-cs-09-1706-s002.zip › NonDemented/32 (11).jpg]

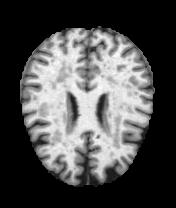

Supplement: Supplemental Information 2 [file peerj-cs-09-1706-s002.zip › NonDemented/31 (18).jpg]

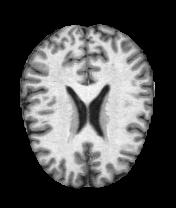

Supplement: Supplemental Information 2 [file peerj-cs-09-1706-s002.zip › NonDemented/26 (78).jpg]

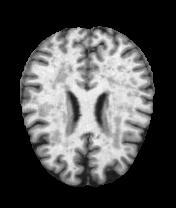

Supplement: Supplemental Information 2 [file peerj-cs-09-1706-s002.zip › NonDemented/30 (18).jpg]

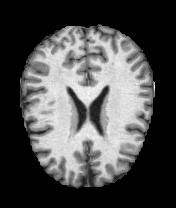

Supplement: Supplemental Information 2 [file peerj-cs-09-1706-s002.zip › NonDemented/27 (78).jpg]

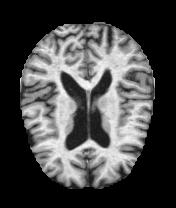

Supplement: Supplemental Information 2 [file peerj-cs-09-1706-s002.zip › NonDemented/nonDem2026.jpg]

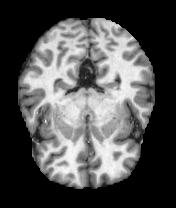

Supplement: Supplemental Information 2 [file peerj-cs-09-1706-s002.zip › NonDemented/nonDem27.jpg]

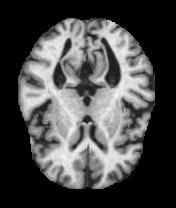

Supplement: Supplemental Information 2 [file peerj-cs-09-1706-s002.zip › NonDemented/nonDem506.jpg]

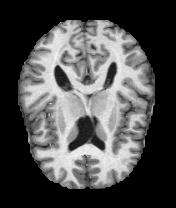

Supplement: Supplemental Information 2 [file peerj-cs-09-1706-s002.zip › NonDemented/nonDem1249.jpg]

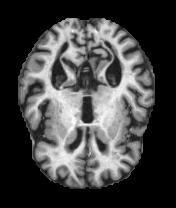

Supplement: Supplemental Information 2 [file peerj-cs-09-1706-s002.zip › NonDemented/nonDem260.jpg]

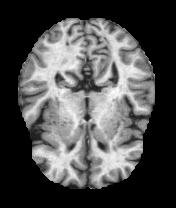

Supplement: Supplemental Information 2 [file peerj-cs-09-1706-s002.zip › NonDemented/nonDem274.jpg]

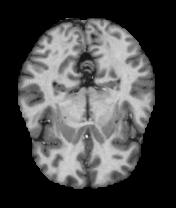

Supplement: Supplemental Information 2 [file peerj-cs-09-1706-s002.zip › NonDemented/nonDem33.jpg]

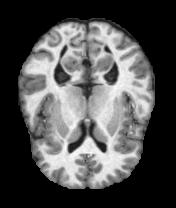

Supplement: Supplemental Information 2 [file peerj-cs-09-1706-s002.zip › NonDemented/nonDem512.jpg]

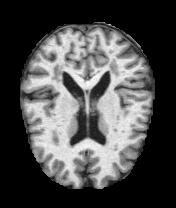

Supplement: Supplemental Information 2 [file peerj-cs-09-1706-s002.zip › NonDemented/nonDem2032.jpg]

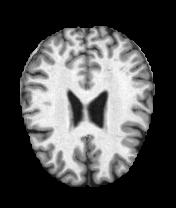

Supplement: Supplemental Information 2 [file peerj-cs-09-1706-s002.zip › NonDemented/29 (40).jpg]

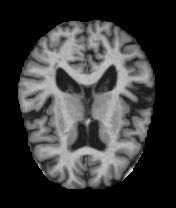

Supplement: Supplemental Information 2 [file peerj-cs-09-1706-s002.zip › NonDemented/nonDem1513.jpg]

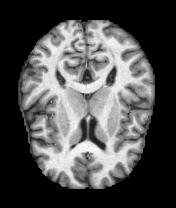

Supplement: Supplemental Information 2 [file peerj-cs-09-1706-s002.zip › NonDemented/nonDem1275.jpg]

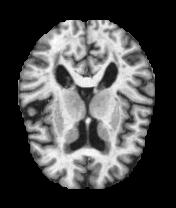

Supplement: Supplemental Information 2 [file peerj-cs-09-1706-s002.zip › NonDemented/nonDem1261.jpg]

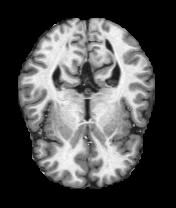

Supplement: Supplemental Information 2 [file peerj-cs-09-1706-s002.zip › NonDemented/nonDem248.jpg]

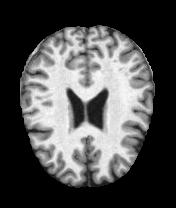

Supplement: Supplemental Information 2 [file peerj-cs-09-1706-s002.zip › NonDemented/28 (40).jpg]

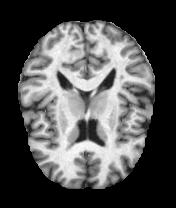

Supplement: Supplemental Information 2 [file peerj-cs-09-1706-s002.zip › NonDemented/nonDem1507.jpg]

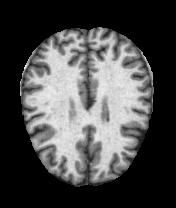

Supplement: Supplemental Information 2 [file peerj-cs-09-1706-s002.zip › NonDemented/32 (85).jpg]

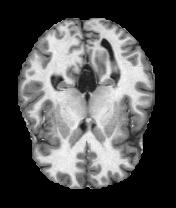

Supplement: Supplemental Information 2 [file peerj-cs-09-1706-s002.zip › NonDemented/nonDem249.jpg]

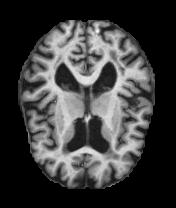

Supplement: Supplemental Information 2 [file peerj-cs-09-1706-s002.zip › NonDemented/nonDem1260.jpg]

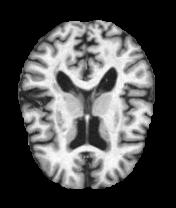

Supplement: Supplemental Information 2 [file peerj-cs-09-1706-s002.zip › NonDemented/nonDem1506.jpg]

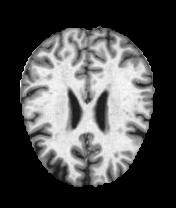

Supplement: Supplemental Information 2 [file peerj-cs-09-1706-s002.zip › NonDemented/31 (63).jpg]

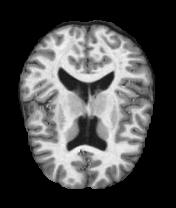

Supplement: Supplemental Information 2 [file peerj-cs-09-1706-s002.zip › NonDemented/nonDem1512.jpg]

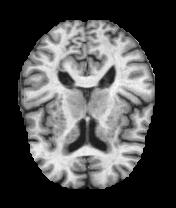

Supplement: Supplemental Information 2 [file peerj-cs-09-1706-s002.zip › NonDemented/nonDem1274.jpg]

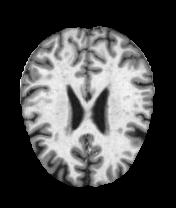

Supplement: Supplemental Information 2 [file peerj-cs-09-1706-s002.zip › NonDemented/30 (63).jpg]

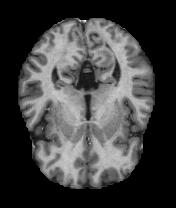

Supplement: Supplemental Information 2 [file peerj-cs-09-1706-s002.zip › NonDemented/nonDem275.jpg]

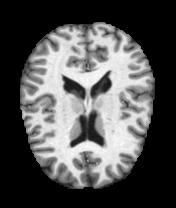

Supplement: Supplemental Information 2 [file peerj-cs-09-1706-s002.zip › NonDemented/nonDem2033.jpg]

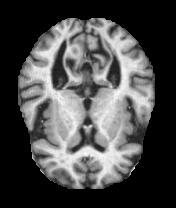

Supplement: Supplemental Information 2 [file peerj-cs-09-1706-s002.zip › NonDemented/nonDem513.jpg]

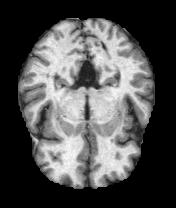

Supplement: Supplemental Information 2 [file peerj-cs-09-1706-s002.zip › NonDemented/nonDem32.jpg]

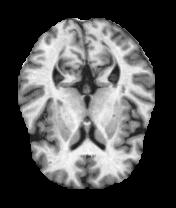

Supplement: Supplemental Information 2 [file peerj-cs-09-1706-s002.zip › NonDemented/nonDem507.jpg]

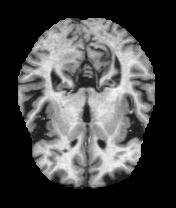

Supplement: Supplemental Information 2 [file peerj-cs-09-1706-s002.zip › NonDemented/nonDem26.jpg]

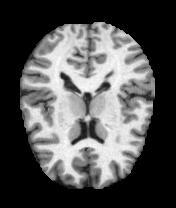

Supplement: Supplemental Information 2 [file peerj-cs-09-1706-s002.zip › NonDemented/nonDem2027.jpg]

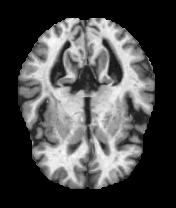

Supplement: Supplemental Information 2 [file peerj-cs-09-1706-s002.zip › NonDemented/nonDem261.jpg]

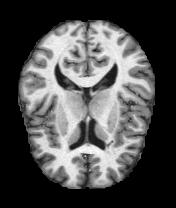

Supplement: Supplemental Information 2 [file peerj-cs-09-1706-s002.zip › NonDemented/nonDem1248.jpg]

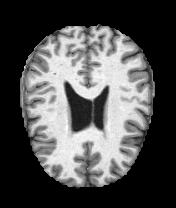

Supplement: Supplemental Information 2 [file peerj-cs-09-1706-s002.zip › NonDemented/27 (54).jpg]

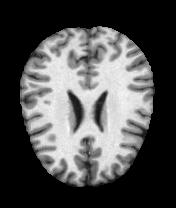

Supplement: Supplemental Information 2 [file peerj-cs-09-1706-s002.zip › NonDemented/30 (34).jpg]

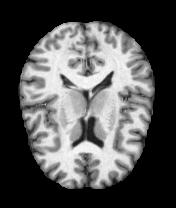

Supplement: Supplemental Information 2 [file peerj-cs-09-1706-s002.zip › NonDemented/nonDem1704.jpg]

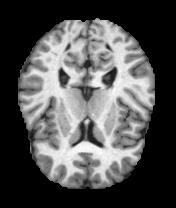

Supplement: Supplemental Information 2 [file peerj-cs-09-1706-s002.zip › NonDemented/nonDem1062.jpg]

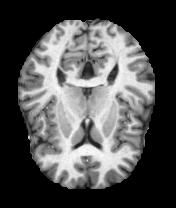

Supplement: Supplemental Information 2 [file peerj-cs-09-1706-s002.zip › NonDemented/nonDem1076.jpg]

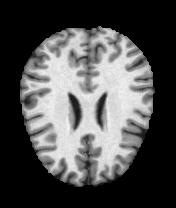

Supplement: Supplemental Information 2 [file peerj-cs-09-1706-s002.zip › NonDemented/31 (34).jpg]

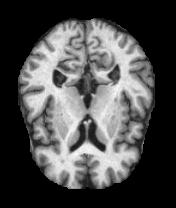

Supplement: Supplemental Information 2 [file peerj-cs-09-1706-s002.zip › NonDemented/nonDem739.jpg]

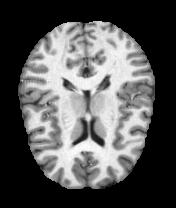

Supplement: Supplemental Information 2 [file peerj-cs-09-1706-s002.zip › NonDemented/nonDem1710.jpg]

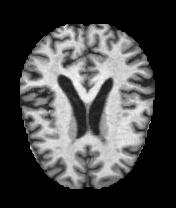

Supplement: Supplemental Information 2 [file peerj-cs-09-1706-s002.zip › NonDemented/nonDem2219.jpg]

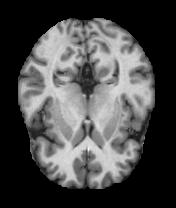

Supplement: Supplemental Information 2 [file peerj-cs-09-1706-s002.zip › NonDemented/nonDem711.jpg]

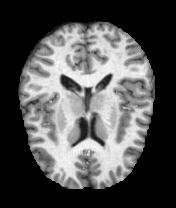

Supplement: Supplemental Information 2 [file peerj-cs-09-1706-s002.zip › NonDemented/nonDem1738.jpg]

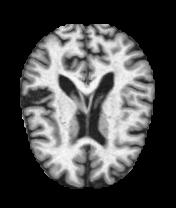

Supplement: Supplemental Information 2 [file peerj-cs-09-1706-s002.zip › NonDemented/nonDem2231.jpg]

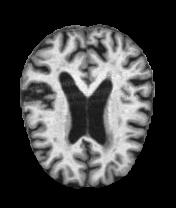

Supplement: Supplemental Information 2 [file peerj-cs-09-1706-s002.zip › NonDemented/nonDem2557.jpg]

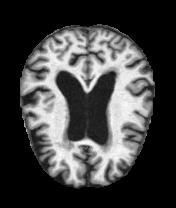

Supplement: Supplemental Information 2 [file peerj-cs-09-1706-s002.zip › NonDemented/nonDem2543.jpg]

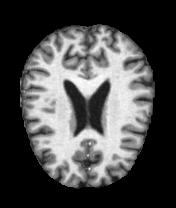

Supplement: Supplemental Information 2 [file peerj-cs-09-1706-s002.zip › NonDemented/26 (100).jpg]

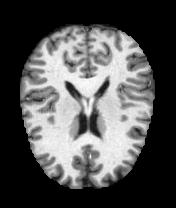

Supplement: Supplemental Information 2 [file peerj-cs-09-1706-s002.zip › NonDemented/nonDem2225.jpg]

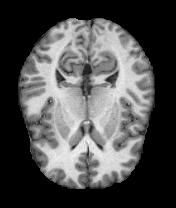

Supplement: Supplemental Information 2 [file peerj-cs-09-1706-s002.zip › NonDemented/nonDem705.jpg]

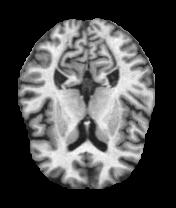

Supplement: Supplemental Information 2 [file peerj-cs-09-1706-s002.zip › NonDemented/nonDem936.jpg]

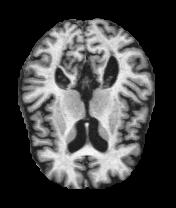

Supplement: Supplemental Information 2 [file peerj-cs-09-1706-s002.zip › NonDemented/nonDem922.jpg]

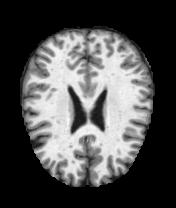

Supplement: Supplemental Information 2 [file peerj-cs-09-1706-s002.zip › NonDemented/29 (83).jpg]

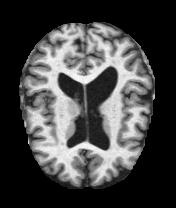

Supplement: Supplemental Information 2 [file peerj-cs-09-1706-s002.zip › NonDemented/nonDem1923.jpg]

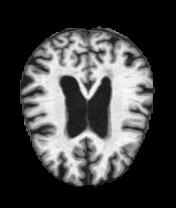

Supplement: Supplemental Information 2 [file peerj-cs-09-1706-s002.zip › NonDemented/31 (5).jpg]

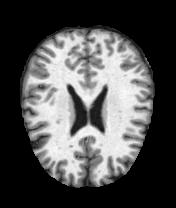

Supplement: Supplemental Information 2 [file peerj-cs-09-1706-s002.zip › NonDemented/28 (83).jpg]

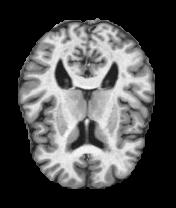

Supplement: Supplemental Information 2 [file peerj-cs-09-1706-s002.zip › NonDemented/nonDem1089.jpg]
