# Supplementary figures and images for: AMSF: attention-based multi-view slice fusion for early diagnosis of Alzheimer’s disease (part 3 of 4)
Source: PeerJ Comput Sci. 2023 Nov 23;9:e1706. doi: 10.7717/peerj-cs.1706 (PMC10703093; doi:10.7717/peerj-cs.1706)

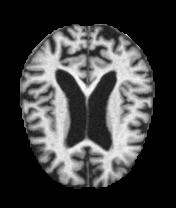

Supplement: Supplemental Information 2 [file peerj-cs-09-1706-s002.zip › ModerateDemented/moderateDem48.jpg]

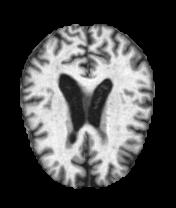

Supplement: Supplemental Information 2 [file peerj-cs-09-1706-s002.zip › ModerateDemented/moderateDem49.jpg]

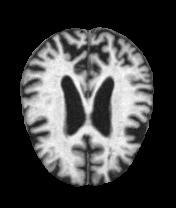

Supplement: Supplemental Information 2 [file peerj-cs-09-1706-s002.zip › ModerateDemented/32 (2).jpg]

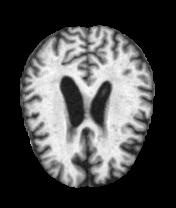

Supplement: Supplemental Information 2 [file peerj-cs-09-1706-s002.zip › ModerateDemented/28.jpg]

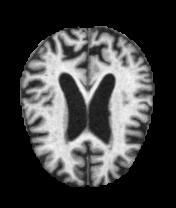

Supplement: Supplemental Information 2 [file peerj-cs-09-1706-s002.zip › ModerateDemented/29 (2).jpg]

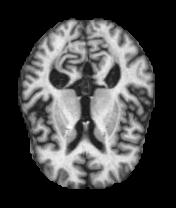

Supplement: Supplemental Information 2 [file peerj-cs-09-1706-s002.zip › ModerateDemented/moderateDem17.jpg]

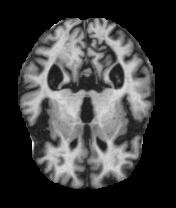

Supplement: Supplemental Information 2 [file peerj-cs-09-1706-s002.zip › ModerateDemented/moderateDem2.jpg]

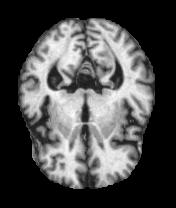

Supplement: Supplemental Information 2 [file peerj-cs-09-1706-s002.zip › ModerateDemented/moderateDem3.jpg]

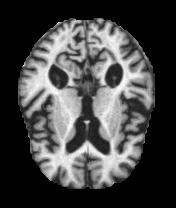

Supplement: Supplemental Information 2 [file peerj-cs-09-1706-s002.zip › ModerateDemented/moderateDem16.jpg]

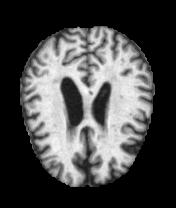

Supplement: Supplemental Information 2 [file peerj-cs-09-1706-s002.zip › ModerateDemented/29.jpg]

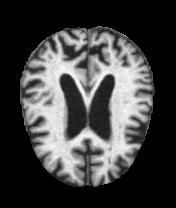

Supplement: Supplemental Information 2 [file peerj-cs-09-1706-s002.zip › ModerateDemented/30 (2).jpg]

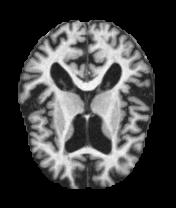

Supplement: Supplemental Information 2 [file peerj-cs-09-1706-s002.zip › ModerateDemented/moderateDem28.jpg]

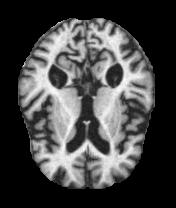

Supplement: Supplemental Information 2 [file peerj-cs-09-1706-s002.zip › ModerateDemented/moderateDem14.jpg]

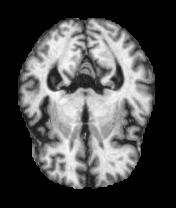

Supplement: Supplemental Information 2 [file peerj-cs-09-1706-s002.zip › ModerateDemented/moderateDem1.jpg]

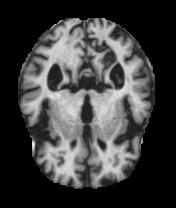

Supplement: Supplemental Information 2 [file peerj-cs-09-1706-s002.zip › ModerateDemented/moderateDem0.jpg]

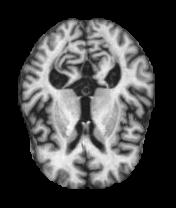

Supplement: Supplemental Information 2 [file peerj-cs-09-1706-s002.zip › ModerateDemented/moderateDem15.jpg]

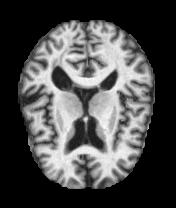

Supplement: Supplemental Information 2 [file peerj-cs-09-1706-s002.zip › ModerateDemented/moderateDem29.jpg]

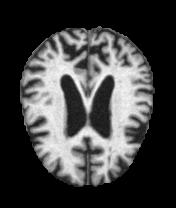

Supplement: Supplemental Information 2 [file peerj-cs-09-1706-s002.zip › ModerateDemented/31 (2).jpg]

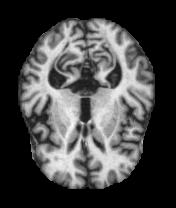

Supplement: Supplemental Information 2 [file peerj-cs-09-1706-s002.zip › ModerateDemented/moderateDem11.jpg]

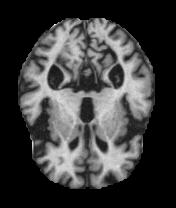

Supplement: Supplemental Information 2 [file peerj-cs-09-1706-s002.zip › ModerateDemented/moderateDem4.jpg]

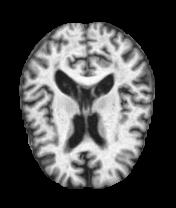

Supplement: Supplemental Information 2 [file peerj-cs-09-1706-s002.zip › ModerateDemented/moderateDem39.jpg]

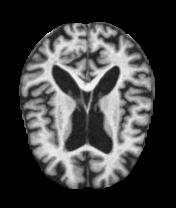

Supplement: Supplemental Information 2 [file peerj-cs-09-1706-s002.zip › ModerateDemented/moderateDem38.jpg]

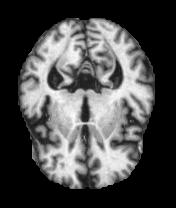

Supplement: Supplemental Information 2 [file peerj-cs-09-1706-s002.zip › ModerateDemented/moderateDem5.jpg]

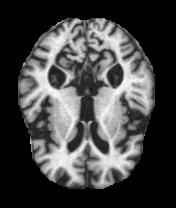

Supplement: Supplemental Information 2 [file peerj-cs-09-1706-s002.zip › ModerateDemented/moderateDem10.jpg]

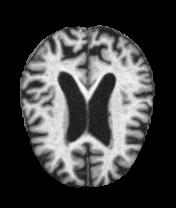

Supplement: Supplemental Information 2 [file peerj-cs-09-1706-s002.zip › ModerateDemented/28 (2).jpg]

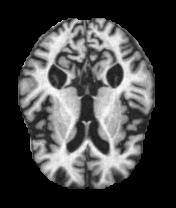

Supplement: Supplemental Information 2 [file peerj-cs-09-1706-s002.zip › ModerateDemented/moderateDem12.jpg]

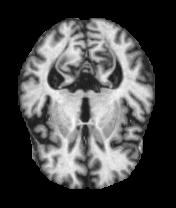

Supplement: Supplemental Information 2 [file peerj-cs-09-1706-s002.zip › ModerateDemented/moderateDem7.jpg]

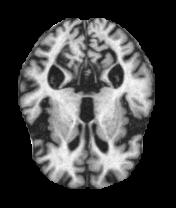

Supplement: Supplemental Information 2 [file peerj-cs-09-1706-s002.zip › ModerateDemented/moderateDem6.jpg]

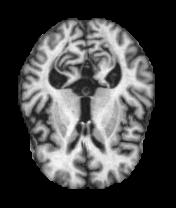

Supplement: Supplemental Information 2 [file peerj-cs-09-1706-s002.zip › ModerateDemented/moderateDem13.jpg]

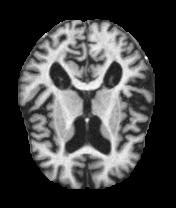

Supplement: Supplemental Information 2 [file peerj-cs-09-1706-s002.zip › ModerateDemented/moderateDem22.jpg]

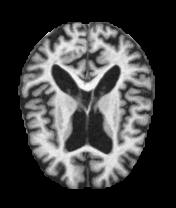

Supplement: Supplemental Information 2 [file peerj-cs-09-1706-s002.zip › ModerateDemented/moderateDem36.jpg]

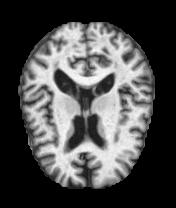

Supplement: Supplemental Information 2 [file peerj-cs-09-1706-s002.zip › ModerateDemented/moderateDem37.jpg]

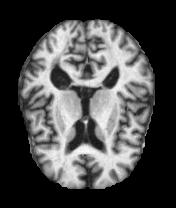

Supplement: Supplemental Information 2 [file peerj-cs-09-1706-s002.zip › ModerateDemented/moderateDem23.jpg]

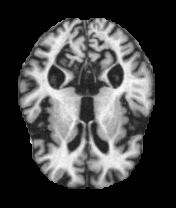

Supplement: Supplemental Information 2 [file peerj-cs-09-1706-s002.zip › ModerateDemented/moderateDem8.jpg]

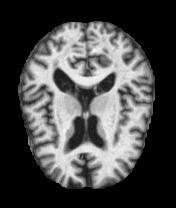

Supplement: Supplemental Information 2 [file peerj-cs-09-1706-s002.zip › ModerateDemented/moderateDem35.jpg]

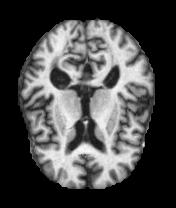

Supplement: Supplemental Information 2 [file peerj-cs-09-1706-s002.zip › ModerateDemented/moderateDem21.jpg]

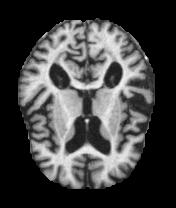

Supplement: Supplemental Information 2 [file peerj-cs-09-1706-s002.zip › ModerateDemented/moderateDem20.jpg]

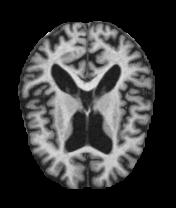

Supplement: Supplemental Information 2 [file peerj-cs-09-1706-s002.zip › ModerateDemented/moderateDem34.jpg]

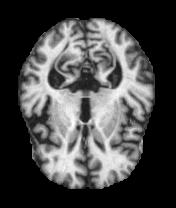

Supplement: Supplemental Information 2 [file peerj-cs-09-1706-s002.zip › ModerateDemented/moderateDem9.jpg]

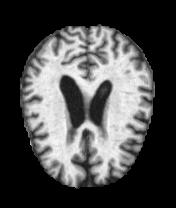

Supplement: Supplemental Information 2 [file peerj-cs-09-1706-s002.zip › ModerateDemented/27.jpg]

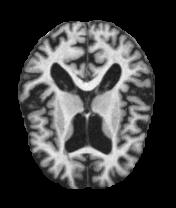

Supplement: Supplemental Information 2 [file peerj-cs-09-1706-s002.zip › ModerateDemented/moderateDem30.jpg]

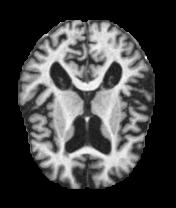

Supplement: Supplemental Information 2 [file peerj-cs-09-1706-s002.zip › ModerateDemented/moderateDem24.jpg]

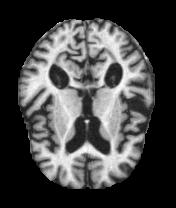

Supplement: Supplemental Information 2 [file peerj-cs-09-1706-s002.zip › ModerateDemented/moderateDem18.jpg]

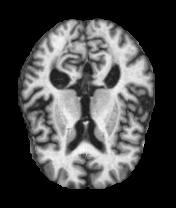

Supplement: Supplemental Information 2 [file peerj-cs-09-1706-s002.zip › ModerateDemented/moderateDem19.jpg]

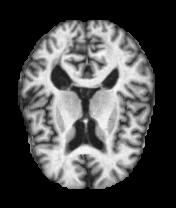

Supplement: Supplemental Information 2 [file peerj-cs-09-1706-s002.zip › ModerateDemented/moderateDem25.jpg]

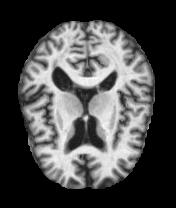

Supplement: Supplemental Information 2 [file peerj-cs-09-1706-s002.zip › ModerateDemented/moderateDem31.jpg]

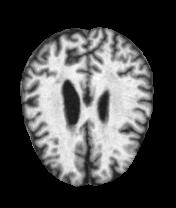

Supplement: Supplemental Information 2 [file peerj-cs-09-1706-s002.zip › ModerateDemented/32.jpg]

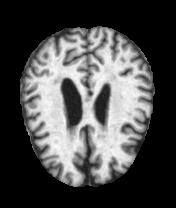

Supplement: Supplemental Information 2 [file peerj-cs-09-1706-s002.zip › ModerateDemented/30.jpg]

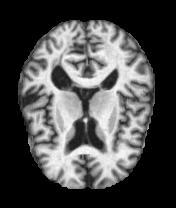

Supplement: Supplemental Information 2 [file peerj-cs-09-1706-s002.zip › ModerateDemented/moderateDem27.jpg]

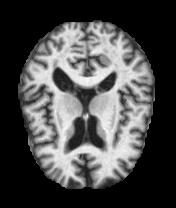

Supplement: Supplemental Information 2 [file peerj-cs-09-1706-s002.zip › ModerateDemented/moderateDem33.jpg]

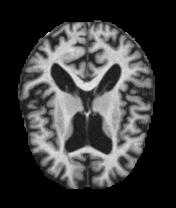

Supplement: Supplemental Information 2 [file peerj-cs-09-1706-s002.zip › ModerateDemented/moderateDem32.jpg]

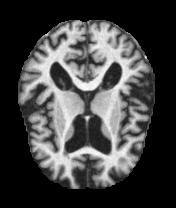

Supplement: Supplemental Information 2 [file peerj-cs-09-1706-s002.zip › ModerateDemented/moderateDem26.jpg]

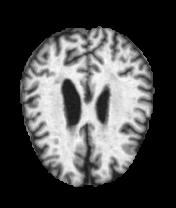

Supplement: Supplemental Information 2 [file peerj-cs-09-1706-s002.zip › ModerateDemented/31.jpg]

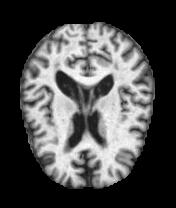

Supplement: Supplemental Information 2 [file peerj-cs-09-1706-s002.zip › ModerateDemented/moderateDem41.jpg]

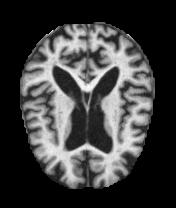

Supplement: Supplemental Information 2 [file peerj-cs-09-1706-s002.zip › ModerateDemented/moderateDem40.jpg]

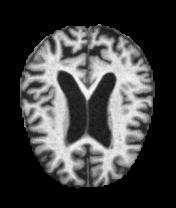

Supplement: Supplemental Information 2 [file peerj-cs-09-1706-s002.zip › ModerateDemented/27 (2).jpg]

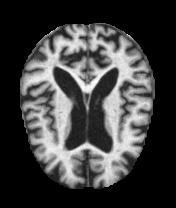

Supplement: Supplemental Information 2 [file peerj-cs-09-1706-s002.zip › ModerateDemented/moderateDem42.jpg]

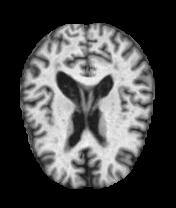

Supplement: Supplemental Information 2 [file peerj-cs-09-1706-s002.zip › ModerateDemented/moderateDem43.jpg]

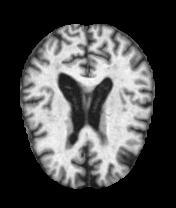

Supplement: Supplemental Information 2 [file peerj-cs-09-1706-s002.zip › ModerateDemented/moderateDem47.jpg]

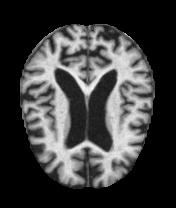

Supplement: Supplemental Information 2 [file peerj-cs-09-1706-s002.zip › ModerateDemented/moderateDem46.jpg]

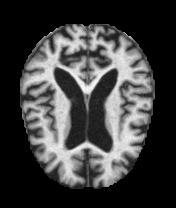

Supplement: Supplemental Information 2 [file peerj-cs-09-1706-s002.zip › ModerateDemented/moderateDem44.jpg]

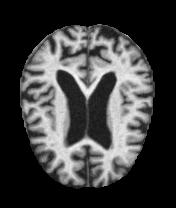

Supplement: Supplemental Information 2 [file peerj-cs-09-1706-s002.zip › ModerateDemented/moderateDem50.jpg]

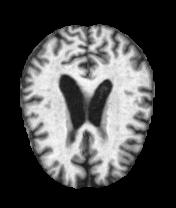

Supplement: Supplemental Information 2 [file peerj-cs-09-1706-s002.zip › ModerateDemented/moderateDem51.jpg]

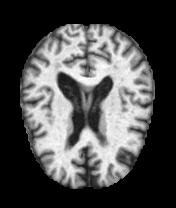

Supplement: Supplemental Information 2 [file peerj-cs-09-1706-s002.zip › ModerateDemented/moderateDem45.jpg]

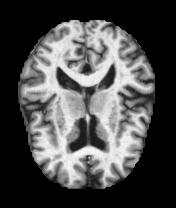

Supplement: Supplemental Information 2 [file peerj-cs-09-1706-s002.zip › NonDemented/nonDem1498.jpg]

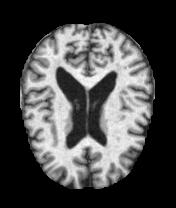

Supplement: Supplemental Information 2 [file peerj-cs-09-1706-s002.zip › NonDemented/nonDem2191.jpg]

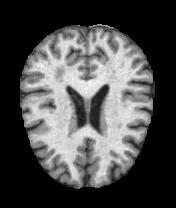

Supplement: Supplemental Information 2 [file peerj-cs-09-1706-s002.zip › NonDemented/26 (81).jpg]

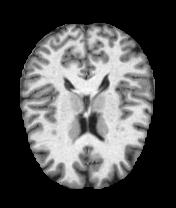

Supplement: Supplemental Information 2 [file peerj-cs-09-1706-s002.zip › NonDemented/nonDem2185.jpg]

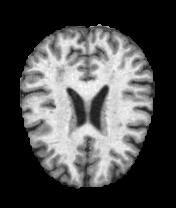

Supplement: Supplemental Information 2 [file peerj-cs-09-1706-s002.zip › NonDemented/27 (81).jpg]

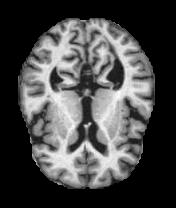

Supplement: Supplemental Information 2 [file peerj-cs-09-1706-s002.zip › NonDemented/nonDem499.jpg]

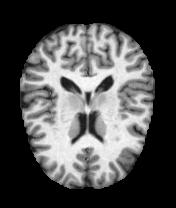

Supplement: Supplemental Information 2 [file peerj-cs-09-1706-s002.zip › NonDemented/nonDem2152.jpg]

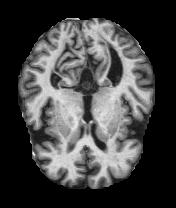

Supplement: Supplemental Information 2 [file peerj-cs-09-1706-s002.zip › NonDemented/nonDem472.jpg]

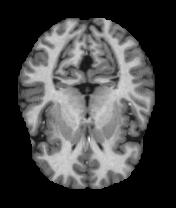

Supplement: Supplemental Information 2 [file peerj-cs-09-1706-s002.zip › NonDemented/nonDem314.jpg]

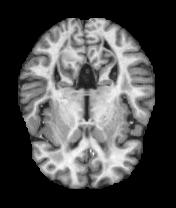

Supplement: Supplemental Information 2 [file peerj-cs-09-1706-s002.zip › NonDemented/nonDem300.jpg]

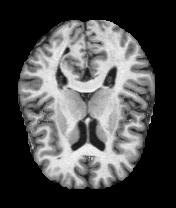

Supplement: Supplemental Information 2 [file peerj-cs-09-1706-s002.zip › NonDemented/nonDem1329.jpg]

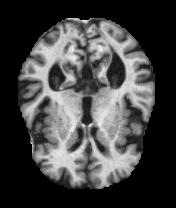

Supplement: Supplemental Information 2 [file peerj-cs-09-1706-s002.zip › NonDemented/nonDem466.jpg]

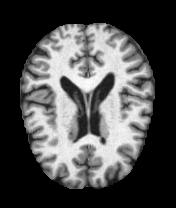

Supplement: Supplemental Information 2 [file peerj-cs-09-1706-s002.zip › NonDemented/nonDem2146.jpg]

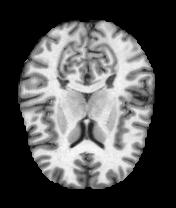

Supplement: Supplemental Information 2 [file peerj-cs-09-1706-s002.zip › NonDemented/nonDem1467.jpg]

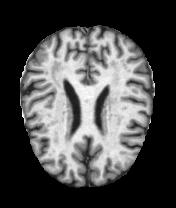

Supplement: Supplemental Information 2 [file peerj-cs-09-1706-s002.zip › NonDemented/28 (56).jpg]

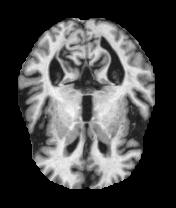

Supplement: Supplemental Information 2 [file peerj-cs-09-1706-s002.zip › NonDemented/nonDem328.jpg]

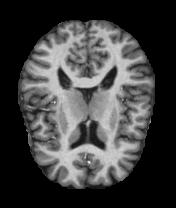

Supplement: Supplemental Information 2 [file peerj-cs-09-1706-s002.zip › NonDemented/nonDem1301.jpg]

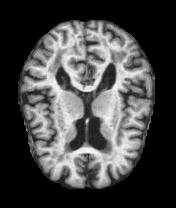

Supplement: Supplemental Information 2 [file peerj-cs-09-1706-s002.zip › NonDemented/nonDem1315.jpg]

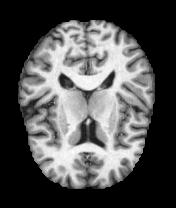

Supplement: Supplemental Information 2 [file peerj-cs-09-1706-s002.zip › NonDemented/nonDem1473.jpg]

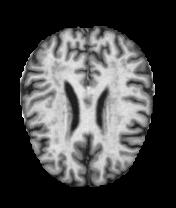

Supplement: Supplemental Information 2 [file peerj-cs-09-1706-s002.zip › NonDemented/29 (56).jpg]

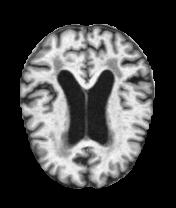

Supplement: Supplemental Information 2 [file peerj-cs-09-1706-s002.zip › NonDemented/nonDem2393.jpg]

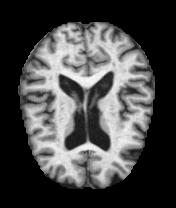

Supplement: Supplemental Information 2 [file peerj-cs-09-1706-s002.zip › NonDemented/nonDem1842.jpg]

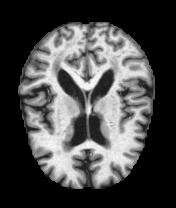

Supplement: Supplemental Information 2 [file peerj-cs-09-1706-s002.zip › NonDemented/nonDem1856.jpg]

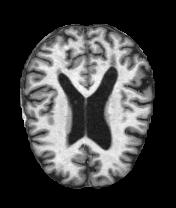

Supplement: Supplemental Information 2 [file peerj-cs-09-1706-s002.zip › NonDemented/nonDem2387.jpg]

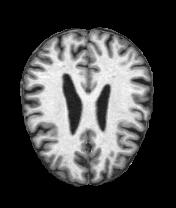

Supplement: Supplemental Information 2 [file peerj-cs-09-1706-s002.zip › NonDemented/32 (50).jpg]

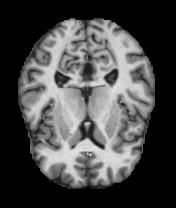

Supplement: Supplemental Information 2 [file peerj-cs-09-1706-s002.zip › NonDemented/nonDem857.jpg]

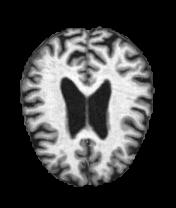

Supplement: Supplemental Information 2 [file peerj-cs-09-1706-s002.zip › NonDemented/31 (59).jpg]

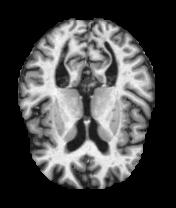

Supplement: Supplemental Information 2 [file peerj-cs-09-1706-s002.zip › NonDemented/nonDem843.jpg]

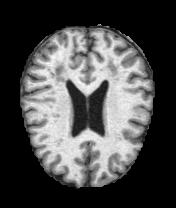

Supplement: Supplemental Information 2 [file peerj-cs-09-1706-s002.zip › NonDemented/27 (39).jpg]

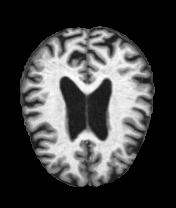

Supplement: Supplemental Information 2 [file peerj-cs-09-1706-s002.zip › NonDemented/30 (59).jpg]

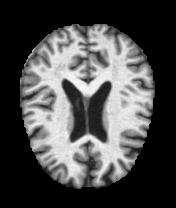

Supplement: Supplemental Information 2 [file peerj-cs-09-1706-s002.zip › NonDemented/nonDem2436.jpg]

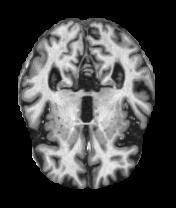

Supplement: Supplemental Information 2 [file peerj-cs-09-1706-s002.zip › NonDemented/nonDem116.jpg]

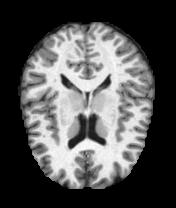

Supplement: Supplemental Information 2 [file peerj-cs-09-1706-s002.zip › NonDemented/nonDem1881.jpg]

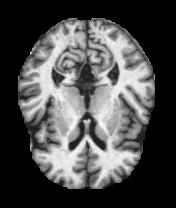

Supplement: Supplemental Information 2 [file peerj-cs-09-1706-s002.zip › NonDemented/nonDem670.jpg]

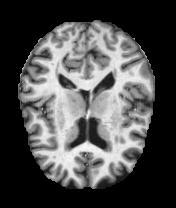

Supplement: Supplemental Information 2 [file peerj-cs-09-1706-s002.zip › NonDemented/nonDem1659.jpg]

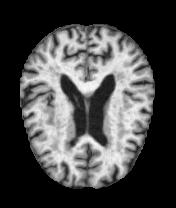

Supplement: Supplemental Information 2 [file peerj-cs-09-1706-s002.zip › NonDemented/nonDem2350.jpg]
